# A Facile and Reproducible Method for the Purification of Peptide- and Protein-Functionalized DNA Nanostructures

**DOI:** 10.1021/jacsau.5c01209

**Published:** 2025-11-14

**Authors:** Izar Schärf, Anna Paton, Betül Karakoc, Bianca Greul, Anna Scheeder, Paraskevi Nani, Pol Saludes Peris, Maria Zacharopoulou, Viktorija Glembockyte, Ioanna Mela

**Affiliations:** † Department of Pharmacology, 2152 University of Cambridge, Tennis Court Road, Cambridge CB2 1PD, U.K.; ‡ Institute for Molecular Systems Engineering and Advanced Materials, 9144 Universitat Heidelberg, INF 225, 69120 Heidelberg, Germany; § Department of Biomedical Engineering, University of Strathclyde, 106 Rottenrow East, Glasgow G4 0NW, U.K.; ∥ 28296 Max Planck Institute for Medical Research, Jahnstr. 29, 69120 Heidelberg, Germany; ⊥ Department of Chemical Engineering and Biotechnology, University of Cambridge, Philippa Fawcett Drive, Cambridge CB1 0AS, U.K.

**Keywords:** DNA Nanotechnology, Purification, Size Exclusion Chromatography (SEC), Peptide, Protein, Functionalization

## Abstract

DNA nanotechnology has emerged as a promising field for biomedical applications, in both the therapeutic and diagnostic domains. The ability of DNA nanostructures to carry cargos in precise numbers and orientations makes them competitive candidates for drug delivery, biosensors, or imaging agents. Two of the main challenges for translating DNA nanostructures from the laboratory to the clinic are achieving cost-effective large-scale production and establishing comprehensive safety profiles. Having the ability to reliably and efficiently purify functionalized DNA nanostructures is key to both challenges and an open question in the field of DNA nanotechnology. Here we present a scalable method for the fast and efficient purification of a high concentration of peptide- or protein-functionalized DNA nanostructures. We use a gravity-driven size exclusion chromatography approach that has the potential to purify DNA nanostructures within 10 min in yields of up to 93% with purities of over 99.9% and is appropriate for both protein and peptide conjugates.

## Graphical abstract

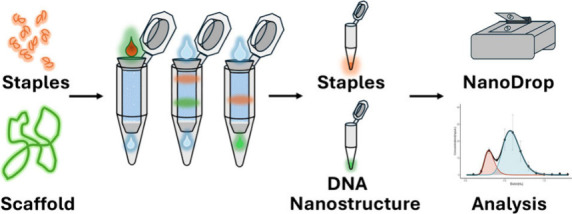

DNA nanotechnology offers a virtually limitless design space for creating nanoscale structures that are generating growing interest for biomedical applications, especially in the diagnostic and therapeutic delivery fields.^1–5^ This can be attributed to their broad application potential, declining cost, and improved design tools.^4,6^ One distinct advantage of DNA nanotechnology over other types of nanoparticles is the ability to orthogonally introduce multiple active payloads such as proteins, peptides, or small molecules on the nanostructures while being able to precisely control their position, orientation, and number of copies.^7–11^ Biomedical applications of DNA nanotechnology have been well-reviewed^6,12–15^ and span a wide range, including synthetic cells, nanomotors, nanopores, bispecifics, catalytic and enzymatic scaffolds, and viral capture systems as well as drug and gene delivery systems.^16–23^

Achieving high purity of functionalized DNA nanostructures is critical not only to ensure that they perform reliably in biomedical applications but also to achieve regulatory approval.^24^ Currently, purification of DNA nanostructures decorated with functional molecules (usually antibodies and peptides) in a way that is purity-, yield-, time-, and cost-efficient can be a challenge.^10,21,24–30^ Although solutions for specific applications have been explored, a universal approach to functionalized DNA nanostructure purification, similar to the widely adopted kits and workflows for DNA/RNA extraction or the robust chromatographic solutions (e.g., Ni-NTA HisTag, biotin affinity, size exclusion chromatography (SEC), or ion exchange) commonly used in protein purification, has yet to be developed.^31^

Existing methods for purifying DNA nanostructures can be inappropriate for those functionalized for biomedical applications. For example, ethanol, poly­(ethylene glycol) (PEG), and ammonium sulfate precipitation methods are limited to DNA nanostructures with small-molecule or oligonucleotide modifications and incompatible with purifying more sensitive protein-conjugated structures.^25,27,29,32^ Gel electrophoresis is slow, labor-intensive, skill-dependent, and nonscalable. Molecular weight cutoff (MWCO) filtration is expensive and hard to scale and can lead to structural damage and unpredictable yields, while molecular weight dialysis, though an option, is time-consuming and challenging to optimize. Both are incompatible with larger protein assemblies.^30^ Methods such as fast protein liquid chromatography (FPLC) and density gradient ultracentrifugation can provide effective purification but require expensive equipment, time, and high minimum working DNA concentrations, making them impractical for many laboratories. Centrifugal SEC in spin columns is ineffective at removing larger proteins and might damage nanostructures sensitive to shear forces.^28,33^ These limitations emphasize the need for a purification method that balances efficiency, scalability, and cost without compromising structural integrity. Gravity-driven size exclusion approaches have shown promise, especially for large-scale purifiaction.^34^ In 2024, Ebrahimimojarad et al.^26^ established Sepharose CL-4B-based gravity-driven size exclusion chromatography as a feasible method for DNA origami purification that could overcome some of the previously mentioned limitations. The method showed good results for protein-decorated nanostructures but is better suited for high quantities of DNA nanostructures.

In this work, we elaborate on this approach and present a gravity-driven size exclusion method that is designed to be practical, fast, efficient, and cost-effective, targeting the small- to medium-scale range (1–10 μg of DNA) of purifications. In parallel, we propose a rapid, automated approach for the quantification of purification efficiency, supported by an open-access computational tool that allows for the quick quantification of purification yield and purity based on simple NanoDrop measurements. This fills a critical gap, enabling researchers to seamlessly transition from proof-of-concept studies to larger-scale experiments without changing the purification systems. By addressing this gap in the range of scales, our method bridges the needs of early-stage validation and experimental deployment toward biomedical applications. Here we apply our purification method to peptide- and protein-functionalized nanostructures. As a proof of principle, we use TBP, a negatively charged, hydrophilic peptide, and streptavidin, a globular protein with neutral net charge. The method can be easily expanded to different nanostructure/cargo combinations with careful consideration of the appropriate buffer solutions, so that the interactions between the functionalized nanostructures and the size-exclusion resin can be minimized.^35–38^

## Results

The primary focus of this work is to set up and characterize a method for quickly and efficiently purifying protein- and peptide-conjugated DNA nanostructures. We focused on 0.8 mL column volume (CV) spin columns for gravity filtration (G-SEC) using Cytiva SuperSEC resin (Figure 1). The columns are set up in two steps: packing and equilibration (the full protocol is given in the Supporting Information (SI)). First the column is filled with resin to achieve the target volume in approximately 5 min. The resin is equilibrated with 1 CV of the elution buffer of choice for another 5 to 10 min. The purification is performed by adding the sample to the packed column and eluting in *N* fractions with the desired fraction volume (Figure 1). Elution takes as little as 3 min and at most 15 min if more than 1 CV of eluent is used. The optimal loading volume (LV) for the 0.8 mL CV column was found to be 50 μL (Figure S5). To assess the scalability at higher CVs, we also tested 2.5 mL CV (100 μL LV) and 5 mL CV (100 μL LV) columns (Figure S6). Testing the reusability of columns showed no deterioration in performance after 10 consecutive uses (Figure S11).

**Figure 1:**
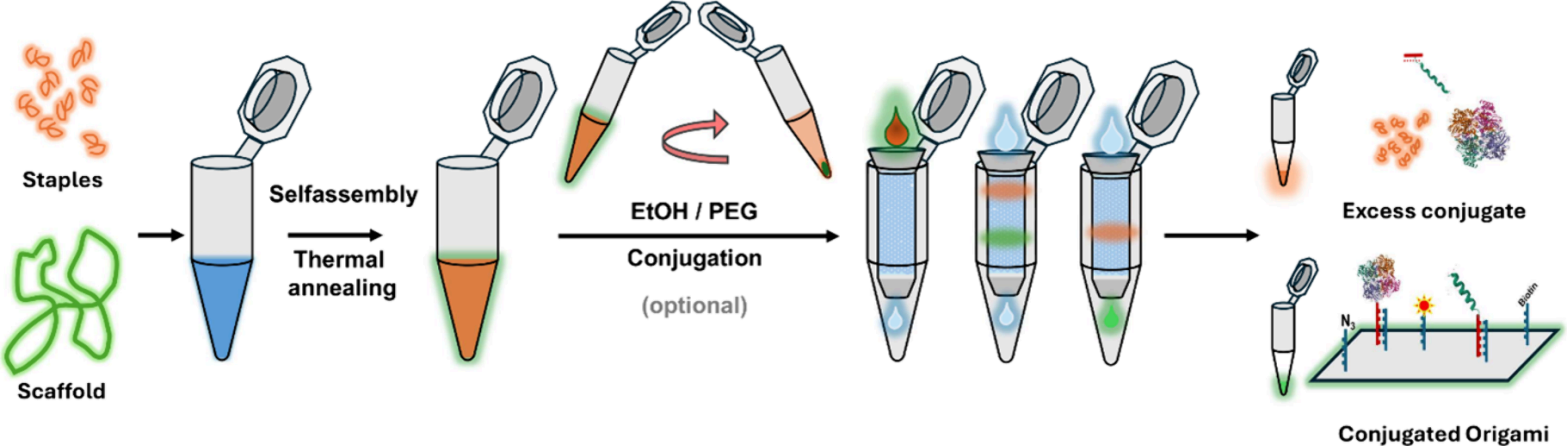
**Workflow for making and purifying DNA nanostructures.** Staple ssDNA and scaffold DNA are mixed and thermally annealed, leading to the self-assembly of the nanostructures. The folded nanostructures can optionally be concentrated by precipitation by centrifugation with EtOH or PEG and then conjugated with the active biomolecules of interest. The functionalized nanostructures are run through the G-SEC column, which separates excess staples and conjugates from the nanostructures.

First, we set out to assess the efficiency of our method in purifying a wide range of unmodified nanostructures, including different 2D (rectangle,^7^ triangle,^39^ 5-well-frame,^40^ frame^41^) and 3D (tetrahedron^42^) geometries (Figure 2). Purification efficiency was assessed using agarose gel electrophoresis to assess the removal of excess staples and NanoDrop measurements to estimate the amount of DNA present in each collected fraction (Figure 2a–c). To further quantify the purification quality, we developed a simple computational approach that allows the use of NanoDrop measurements to calculate the purity and resolution of the collected fractions. These can be obtained by fitting the concentration measurements of each fraction using a sum of two log-normal distributions:

1 $$y=\frac{\alpha_{1}}{x\cdot \sigma_{1}\cdot \sqrt{2\pi }}\cdot \exp\left[-\frac{{\left(\log\left(x\right)-\mu_{1}\right)}^{2}}{2{\sigma_{1}}^{2}}\right]+\frac{\alpha_{2}}{x\cdot \sigma_{2}\cdot \sqrt{2\pi }}\cdot \exp\left[-\frac{{\left(\log\left(x\right)-\mu_{2}\right)}^{2}}{2{\sigma_{2}}^{2}}\right]$$

where *y* is the concentration of an elution fraction as measured by NanoDrop, *x* is the elution volume, μ is the logarithmic mean of the distribution in *x* (elution volume), σ is the standard deviation of the logarithm of the elution volume, and α_1_ and α_2_ are positive scaling constants.

**Figure 2:**
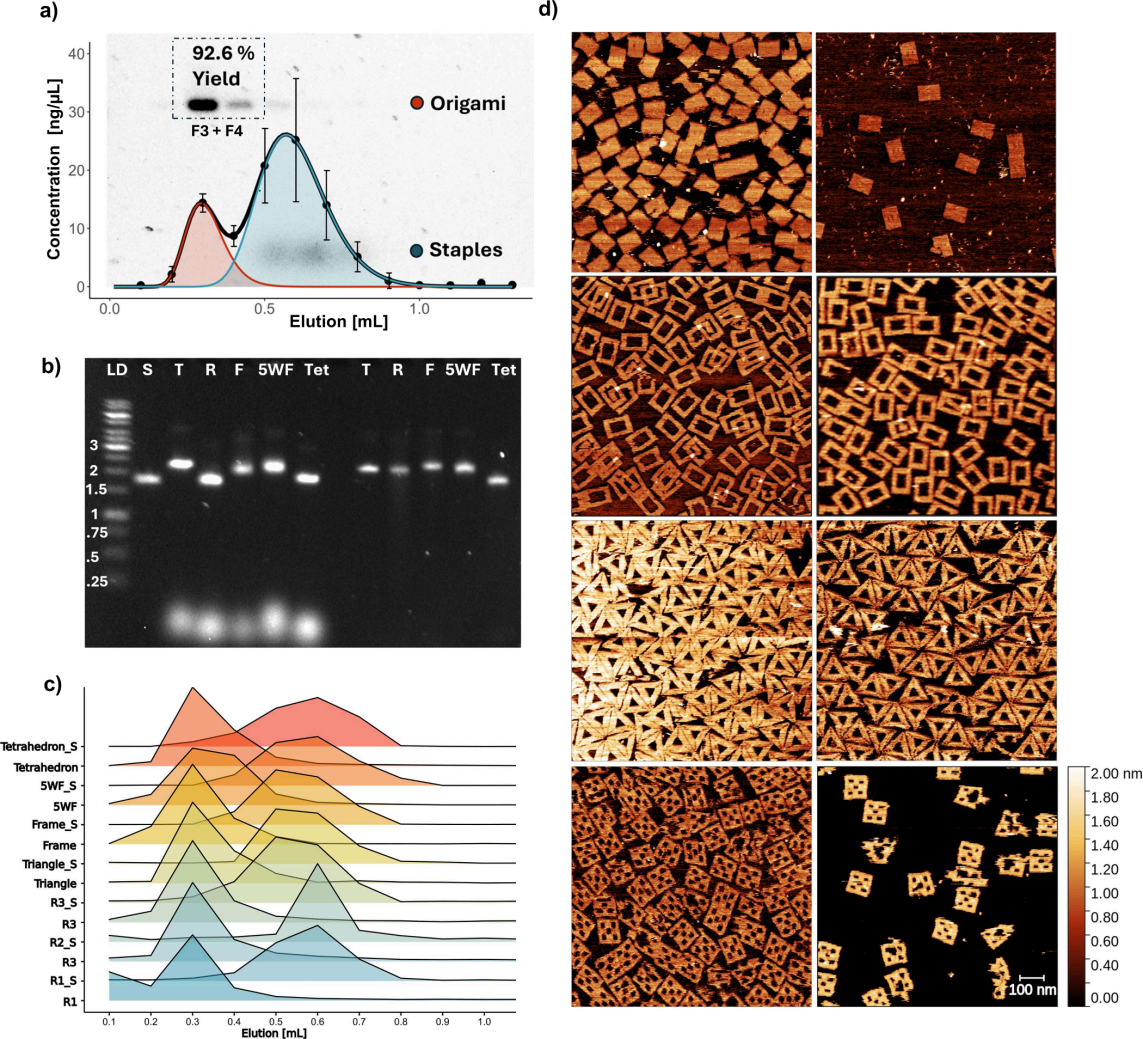
**Characterization of purification efficiency on bare DNA nanostructures.** (a) Concentrations of DNA nanostructures and staple strands measured across seven purification replicates and fitted using the sum of two log-normal probability density functions (eq 1). Error bars denote the standard deviation. The sum is displayed as a black line; the two partial functions are plotted with their AUCs shaded red for DNA nanostructures and blue for staples. (b) Agarose gel electrophoresis of five nanostructures before and after purification (fraction 3): scaffold (S) as a control, triangle (T), rectangle (R), frame (F), 5-well frame (5WF), and 3D tetrahedron (Tet). (c) Normalized band intensities of both DNA nanostructures and excess staples extracted from agarose gel electrophoresis images across fractions and different nanostructures. The intensities of the DNA nanostructures and staples are each normalized to 1. R1 refers to repeat 1 of rectangle until R*n*; R*n*_S refers to the staple bands. (d) Atomic force microscopy images of purified (left) and unpurified (right) DNA nanostructures (rectangle, frame, triangle, and 5-well frame) show efficient purification and no significant damage to the nanostructures.

The resolution is calculated by using the common resolution definition for close peaks, taking the full width at half-maximum approach according to eq 2:

2 $$R_{s}=\frac{2\cdot \left(t_{R2}-t_{R1}\right)}{w_{1/2}^{1}+w_{1/2}^{2}}$$

where *R*_s_ is the resolution, *t*_R*n*_ is the elution volume value at the peak maximum of peak *n*, and *w*_1/2_^*n*^ is the *x* width at half the *y* maximum of peak *n* solved numerically from the fitted partial equations from eq 1.

Traditionally, gel electrophoresis is the only fast and reliable way of analyzing the purity and size distribution of folded DNA nanostructures. FPLC and HPLC with SEC can perform the same analysis; however, they usually have comparatively high requirements for sample concentration, which is not suitable for small-scale test runs. By using the NanoDrop measurements to calculate purity, yield, and resolution of purification, we provide a simple computational tool for quantification of purification efficiency.

Using agarose gel electrophoresis and NanoDrop quantification, we found that elution from a 0.8 mL CV column with 50 μL sample volume and 100 μL fraction volume shows peak elution of nanostructures at 300 μL (fraction 3) and 400 μL (fraction 4) elution volumes (Figure 2a). The combined yield of nanostructures in fractions 3 and 4 measured by NanoDrop is 93 ± 12% of the total mass of DNA nanostructures in the sample with a calculated purity of 72.2 ± 20.2% (*n* = 7), while the individual yield and purity are 57.7 ± 6% and 98.9 ± 1.53%, respectively, for fraction 3 and 35.0 ± 6.5% and 45.4 ± 20.2%, respectively, for fraction 4. The efficient folding of the nanostructures and separation of staple strands from DNA nanostructures are further confirmed by agarose gel electrophoresis (Figure 2b). Applying the same method to five different nanostructures (3D tetrahedron, 5-well frame, frame, triangle, and rectangle), we can calculate the normalized band intensities of each elution fraction, separated into nanostructures and staple strands. Repeating the process three times for the rectangular nanostructure, we demonstrate the reproducibility of the method across and within the nanostructures (Figure 2c). Atomic force microscopy confirms that DNA nanostructures remain intact after purification (74.8 ± 8.4% intact nanostructures before in comparison to 76.3 ± 12.3% after; Figure 2d).

We further explore the applicability of our method to more complex structures using double-layered 3D DNA origami (SI section 4). We show that our purification method works for these nanostructures when unmodified, modified with zinc–porphyrin moieties, and modified with zinc–porphyrin and aptamers in yields of 41.6%, 63.8%, and 34.3%, respectively.

Subsequently, we examined whether our method can be used to reliably purify peptide- and protein-conjugated DNA nanostructures that could have applications in a biomedical context. First, we tested the attachment of a tankyrase-binding peptide (TBP), conjugated to a single-stranded DNA overhang, to DNA triangles that carry complementary DNA “sticky ends”. The peptide is also modified with a C-terminal Cy5 fluorophore for further detection. The DNA nanostructures were first concentrated by ethanol precipitation. We found that the recovery from ethanol precipitation can vary significantly, yielding 79.2 ± 74% (*N* = 6). This variability in the yield can be attributed to the efficiency of pellet recovery upon removal of the supernatant solution. Using larger volumes of starting solution (500 μL of a 10 nM solution of DNA nanostructures (25 μg)) consistently recovers more than 100% of DNA nanostructures’ mass, indicating staple precipitation^10,25,29^ (SI section 3.7.1 and Table S3). This does not pose a problem given the subsequent purification step. Concentrated nanostructures were then incubated with peptides and purified using G-SEC. The efficiency of the conjugation and purification was assessed through agarose gel electrophoresis, Nanodrop measurements, and atomic force microscopy (Figure 3). The yield of functionalized nanostructures was corrected for the yield of the EtOH concentration and was calculated as 64.3 ± 3.5%. The nanostructures predominately elute in fraction 3, while a band that is attributed to nanostructure dimers^43^ is also present.

**Figure 3:**
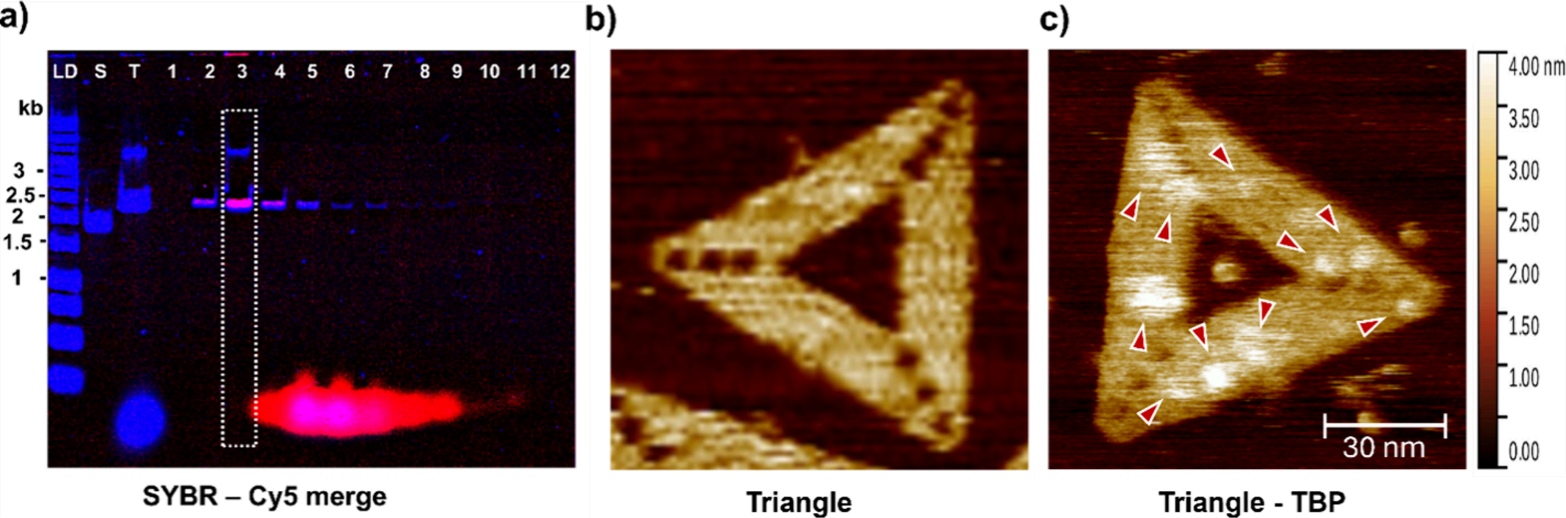
**Purification of peptide-functionalized DNA nanostructures.** (a) Fluorescence image of agarose gel of peptide-functionalized DNA nanostructures. The two channels (SYBR Safe (488), in blue and Cy5 (647), in red) show DNA and peptide, respectively. Fraction 3 is highlighted by the dashed box and shows peptide-loaded nanostructures efficiently separated from excess staples and peptides. (b) AFM of a nonfunctionalized DNA triangle. (c) AFM of a TBP-functionalized DNA nanostructure. Red arrows point to conjugated peptides.

We then assessed the ability of G-SEC to efficiently remove higher-molecular-weight molecules. DNA rectangles were modified with biotinylated staples, and streptavidin was used as a proof-of-principle protein. At the same time, we tested columns with different volumes (0.8, 2.5, and 5 mL) for purification efficiency in this context. For the 0.8 mL column, the recovery yield was 35 ± 4% in fraction 3 and 13 ± 2% in fraction 4, with a combined yield of 48 ± 5%. When corrected for the ethanol concentration step, the yields are 45 ± 6% in fraction 3, 17 ± 3% in fraction 4, and 62 ± 6% in total. Each CV shows clear separation of DNA and protein in the different fractions (Figures 4a and S21–S25). We consistently see the elution of DNA nanostructures in fractions 2–4, while streptavidin elution is visible in fractions 5–9. AFM imaging of purified samples also shows efficient purification of streptavidin-decorated DNA nanostructures (Figures 4b and S19).

**Figure 4:**
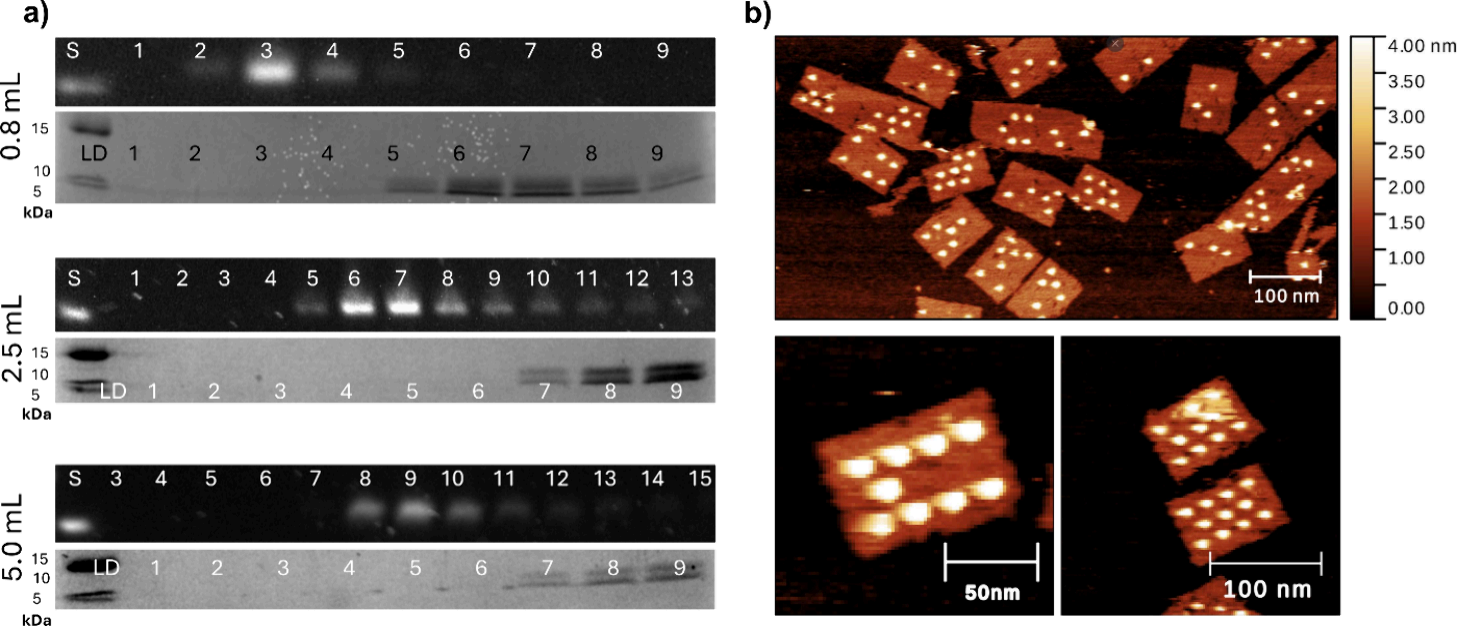
**Purification of protein-functionalized DNA nanostructures.** (a) Purification comparison across three CVs (0.8, 2.5, and 5 mL) shows the ability to separate streptavidin from nanostructures in the aligned elutions of the DNA nanostructures in AGE (top) and streptavidin in SDS-PAGE (bottom). (b) AFM of purified rectangle–streptavidin conjugates eluted in fraction 3 (300 μL EV) on a 0.8 mL CV SuperSEC column.

Finally, we benchmarked G-SEC against existing purification methods using rectangular DNA nanostructures as an example. We tested extraction from agarose gels via continued electrophoresis into sucrose and “FreezeNSqueeze” columns, ultrafiltration via 100 kDa MWCO filters, centrifugal gel filtration with 0.8 mL CV Sephacryl S-300, centrifugal precipitation by ethanol, FPLC-SEC with Superose 6, and G-SEC with Sephacryl S-300 and Cytiva SuperSEC (Figure 5). We compiled the results of the purification test that we performed with values previously reported in the literature to provide an updated comparison of different purification methods used for DNA nanostructures.

**Figure 5:**
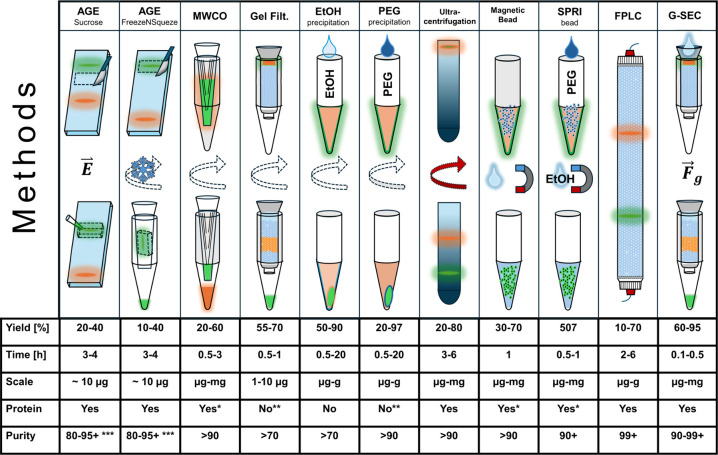
**Functional schematics of the most common methods for DNA nanostructure purification, comparing the yield, time, scale, protein compatibility, and purity.** Ranges are given when the literature suggests different results. E indicates the electric field used to elute the sample, the snowflake indicates freezing, the turning arrows indicate centrifugation (ultracentrifugation in red), the magnets indicate use of magnets, and Fg indicates that gravity was the driving force. *, Preclude sensitive protein conjugates or require modification; **, used for small conjugates; ***, contamination with DNA stain.

## Discussion

We have demonstrated that using 0.8 mL CV G-SEC is a simple, fast (10 min) and efficient (up to 92.6% yield) method for purifying both bare and functionalized DNA nanostructures (64% yield). Our G-SEC method is competitive with methods that are more expensive and time-consuming while also outperforming many in the critical categories of yield and purity. The small G-SEC approach provides a new valuable purification tool for DNA nanostructures functionalized with biologics and peptides, enabling the preparation of conjugates at concentrations and purity suitable for biomedical applications. The gravity-driven nature and the washable resin make it cheap to operate, while using Nanodrop measurements of the collected fractions for analysis of purity and resolution expedites the process further. The fact that the columns are compatible with multiwell plates, reusable, and quick to elute makes them good candidates for automated, high-throughput approaches.

## Supplementary Material



## Notes

Methodology, performance comparison, AFM data, reusability characterization, and code availability (PDF)
