# Supplementary material for: A Facile and Reproducible Method for the Purification of Peptide- and Protein-Functionalized DNA Nanostructures
Source: JACS Au. 2025 Nov 14;5(12):5917–23. doi: 10.1021/jacsau.5c01209 (PMC12728597; doi:10.1021/jacsau.5c01209)
Supplement: Supplementary file 1 [file au5c01209_si_001.pdf]

Supplementary Information
A facile and reproducible method for the purification of peptide- and protein-functionalized DNA nanostructures

Izar Schärf[1,2], Anna Paton[1,3], Betül Karakoc[4], Bianca Greul[1], Anna Scheeder[1,5], Paraskevi Nani[1], Pol Saludes Peris[1], Maria Zacharopoulou[1], Viktorija Glembockyte[4] and Ioanna Mela[1]

[1] Department of Pharmacology, University of Cambridge, Tennis Court Road, CB2 1PD, Cambridge, UK

[2] Institute for Molecular Systems Engineering and Advanced Materials, Universitat Heidelberg, INF 225 Heidelberg, 69120, Germany

[3] Department of Biomedical Engineering, University of Strathclyde, 106 Rottenrow East, Glasgow, G4 0NW

[4]Max Planck Institute for Medical Research, Jahnstr. 29, 69120 Heidelberg, Germany

[5]Department of Chemical Engineering and Biotechnology, Philippa Fawcett Drive, CB1 0AS, Cambridge, UK

Contents

1 G-SEC Purification Method **1**
1.1 Purification Column Setup 1
1.2 Purification 2
1.2.1 Required Materials 2
1.3 Analysis of Results 3
1.4 Tips and Tricks 3

2 Materials and Methods **5**
2.1 Buffers 5
2.2 DNA Nanostrucuture folding 5
2.3 Agrose gel eletrophoresis 5
2.4 NanoDrop 5
2.5 Atomic Force Microscopy 5
2.6 SDS-PAGE 6
2.7 ssDNA peptide conjugation, purification and hybridization on DNA Origami 6
2.8 DNA Nanotructure conjugation 6
2.9 Purification Methods 6
2.10 Function Fitting NanoDrop Data 7

3 Additional Data **9**
3.1 Cytiva S-300 vs SuperSEC performance in 0.8 mL CV 9
3.2 Loading volumes for 0.8 mL CV Column 9
3.3 Column Volumes and Protein Seperation 11
3.4 Nanostrucuture integrety 12
3.5 Purification of different Nanostructures 13
3.6 Reusability 15
3.7 Purification Method Comparison 15
3.7.1 EtOH precipitation - 25μg DNA Origami 17
3.8 Method comparison Literature 18

4 Reproduction in an Independent Laboratory **20**

4.1 Gels and AFM imaging 20

4.2 Yields 23

4.3 Methods 23

4.3.1 DNA Origami Folding 23

4.3.2 AFM imaging 24

4.3.3 Gel imaging 24

5 Quantification of Streptavidin Attachment **25**

6 Code Availabilty **25**

7 Bibliography **25**

8 Addendum **27**

8.1 AFM pre/post purification Streptavidin conjugation 27

8.2 Uncropped Gel images for Figure 4 28

8.3 Intactness counting images 30

1 G-SEC Purification Method

1.1 Purification Column Setup

The stock solution of Cytiva SuperSEC resin was thoroughly resuspended, and 25 mL were transferred into a 50 mL falcon tube, to provide a working suspension. 0.8 mL Column Volume (CV) BioRad Micro Bio-Spin columns were used for the purifications. To pack the columns, the bottom stopper was broken off, and the column was placed in a holding rack with a drip tray or an Eppendorf tube, depending on column size. Care was taken for the column to be set up vertically and not to be disturbed throughout the process. 0.5 mL of resuspended resin was transferred into the column, and the solution was let to drain through. This process was repeated until the desired column volume (CV) was reached. Optionally, the column can be pre-wetted with 30-70% EtOH solution to ease the wetting of the bottom frit.

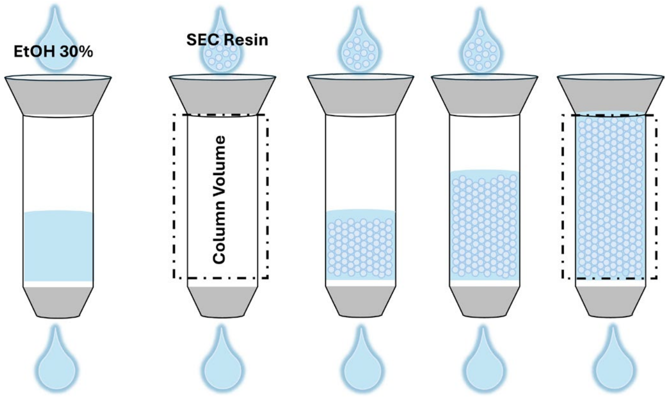

Figure S1: Preparation of a gravity SEC spin column.

1.2 Purification

For purification of freshly folded DNA origami, the column was first equilibrated elution buffer (EB). For the 0.8 mL CV columns 1.5mL EB is used for equilibration, ~2 CV was similarly applied to the 2.5mL and 5mL CV columns. The buffer most commonly used is the same buffer as the one the nanostructures were folded in. If a buffer change is required, using the desired end buffer to equilibrate the column will result in buffer exchange. This sequence allowed for the fastest equilibration of the column, however if substantial buffer exchange is needed, it is advisable to use higher EB volume. All buffers used in the set-up and equilibration process were filtered through a 0.22μm filter.
Once the columns were equilibrated, the sample collection process was set-up: A series of eppendorf tubes, that would collect each purified fraction, were set up on a rack and appropriately labelled. The column was initially placed over the first tube, and the sample was gently pipetted onto the centre of the resin surface. Care was taken to ensure that no excess elution buffer was seen on the surface of the resin, the resin contained no bubbles, and that the resin had settled with a flat top, so that the purification is reproducible, and that resolution is maximized.
When all of the sample volume has run through the column, the elution process starts by adding appropriate volumes of elution fractions to the column. For the 0.8 mL CV columns, it was observed that 100 μL was the optimal volume, as lower volumes might result in slow or poor elution, as well as poor separation. Increasing the elution volume can lead to very dilute samples and worse resolution and separation. When the first fraction has been eluted, the process is repeated until the desired number of fractions is eluted. Typically, DNA nanostructures will elute within the first five fractions (0.5 CV of EB).
For the 0.8 mL CV columns, this should take 5–10 minutes, adding 1.5 mL to a 0.8 mL CV column is best done by filling the 1 mL capacity running buffer space at the top of the spin column with 1 mL EB, then 0.5 mL EB. This ensures the fastest equilibration. If the buffer change is drastic, using higher volumes for equilibration is advisable. If available, use a top frit to keep the resin flat.

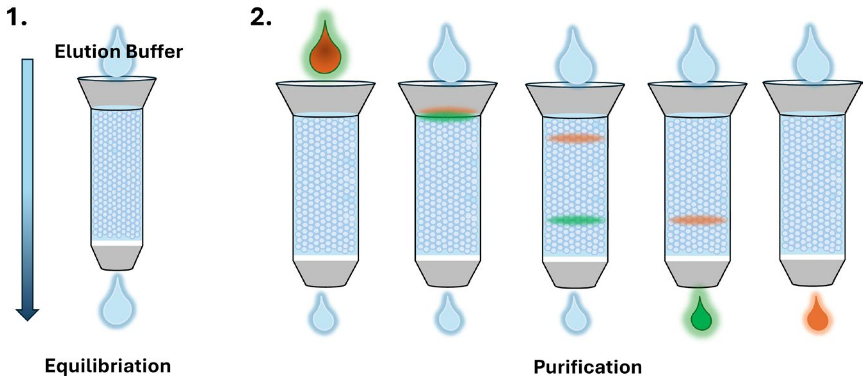

Figure S2: **Process of preparing a column for purification. 1.** The SEC column is equili- brated using the elution buffer. **2.** The sample is applied to the column, and the sample buffer is eluted. Elution buffer is then applied in appropriate volume fractions, and the process is repeated until all desired fractions are collected.

1.2.1 Required Materials

- Cytiva SuperSEC resin
  - Micro Bio-Spin™ Chromatography Columns [#7326204]
  - DNA nanostructures
  - Elution Buffer
  - Eppendorf tubes
  - Pipettes 10-100μL and 0.1-1mL

1.3 Analysis of Results

Analysis can be conducted by measuring the concentrations of each fraction using a spectrometer, as described in section 2.4 and fitting the results as described in section 2.10 to quantify the purity and resolution of each fraction. When the purification process is run for the first time for a new sample, agarose gel electrophoresis should be used to confirm that staple strands or excess proteins and peptides are being separated from the nanostructures and that the nanostructures show a different running behavior to that of the scaffold. An unpurified sample can be run alongside purified fractions to allow for comparison of the pre- and post-purified product. The samples can then be imaged using atomic force microscopy, to assess the correct formation and stability of the DNA nanostructures.

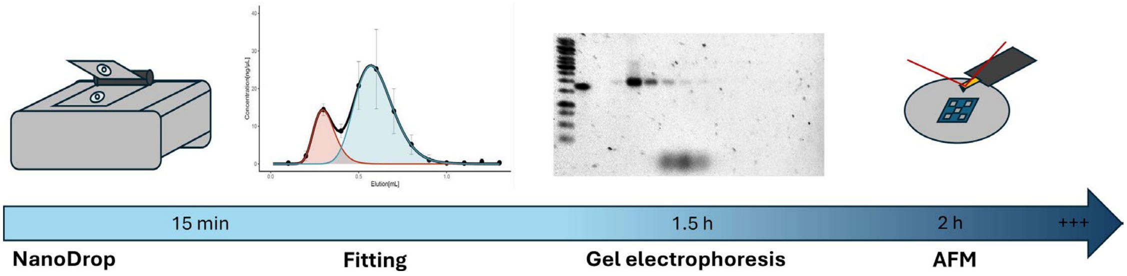

Figure S3: Standard analysis options and workflow-timeline for the purification of DNA nanos- tructures. Nanodrop is the fastest, taking about 1 min per sample in technical triplicates. The following analysis with an existing fitting script similarly takes a few minutes at most. Gel elec- trophoresis usually takes over an hour to complete. AFM can be fast, however, optimizing imaging can take a long time.

1.4 Tips and Tricks

Elution: If the suspension solution does not drain one can force it to go through the frit at the bottom of the spin column by simply placing a lid on the column. This should produce enough pressure to initialize the elution process. If this does not work, one can try a very short spin in a slow benchtop minicentrifuge. These steps should only be performed for the first addition of resin. In further steps, till reaching the desired CV, the resin should be left to settle and drain as vertically and stably as possible.
Bubbles: If a column develops bubbles in the resin, which can happen over time, especially with opening and closing cycles, the column can be re-set by vortexing it upside-down with about 200-400μL of buffer and then turning it right-side up and letting it drain. This should reset the column for further use. This process inevitably results in losing a small amount of resin which means that the column will decrease in CV over time. Please note that this process should not be attempted during fraction elution, but only between experiments.
Setup: Before setting up the column, it can be washed with 30-50% EtOH solution. This will make it easier for the storage buffer of the resin to elute through the column.
Storage: the columns should be stored in the fridge, and if not in very regular use, they should be stored in 30% EtOH as indicated by Cytiva. The best way to store the columns and make sure they do not develop bubbles is by adding buffer to the top of the column and allowing for some buffer to start eluting. Adding the bottom cap, making sure that it is full of eluate and contains no bubbles and then adding the top cap, avoids any bubbles from being forced up through the frit when closing the bottom of the column. To open the column for use first remove the top cap then the bottom cap. For long term storage, the columns can be sealed in a 50mL Falcon tube, filled with 30%EtOH(aq) and sealed with parafilm to prevent the column from drying out.
Cleaning: After purifying potentially sticky proteins, the column can be cleaned using cleaning- in-place procedures. Alternatively, the resin can be collected in 50 mL falcon tubes and washed through centrifugation.

Troubleshooting the buffers: DNA nanostructures will run through the column differently based on the ion concentrations present in the buffer. Using monovalent cation (e.g. Na+/ Cl-) solution in high concentrations may disrupt unwanted interactions with the resin. This might be especially relevant for some protein decorated nanostructures. DNA nanostructures have been found to interact with SEC resin, and this interaction can be reduced by reducing the concentration of Mg2+. The Mg2+ concentration can be safely reduced below 1 mM without compromising the stability of the nanostructures.[1, 2] If necessary, Mg2+ can be reintroduced after purification. The pH of the running buffer can also influence running behavior especially in protein-conjugated samples where the isoelectric point of the attached protein cannot be ignored (for example, if the proteins are naturally positively charged, they might adsorb to the highly negative DNA nanostructures).

2 Materials and Methods

2.1 Buffers

TBE, TAE and TE were prepared according to Cold Spring Harbor protocols with MgCl2 added to 5 mM.[3–5] DNA Origami Buffer was prepared according to Castro et al.[6] The buffer used for protein and peptide conjugated DNA Origami purification by gravity-SEC was 150 mM NaCl 50 mM NaPO4 7 mM MgCl2, pH 7.4. All buffers were filtered either by vacuum filtration or syringe filtration through a 0.22 μm membrane.

2.2 DNA Nanostrucuture folding

DNA nanostructures were folded in a MiniAmp™ Thermal Cycler (ThermoFischer Scientific) by quickly heating the sample to from 90 °C and then slowly cooling it down to 25 °C cooling with a rate of 1 C/min. Folding mix for all types of nanostructures is detailed in Table S1.

reagent	Volume [μL]	Concentration [nM]
MiliQ H_2O	55	-
Staple mix	25	200
M13mp18 ssDNA	10	100
Origami Buffer	10	10X

Table S1: Reagents for folding 100 μL of 10.6 nM DNA Origami

2.3 Agrose gel eletrophoresis

Agarose gel electrophoresis was performed in a BioRad MiniSub Cell GT chamber at 90V for 45-90 min in an ice slurry bath. 50mL of 1% agarose gels were prepared and ran with 1X TBE with 5.5 mM MgCl2 (TrizmaBase Sigma Aldrich) and 5μL SYBR™ Safe DNA Gel Stain (ThermoFischer Scientific Invitrogen). DNA nanostructure sizes were confirmed with the GeneRuler 1kb DNA ladder (ThermoFisher Scientific). Gels were imaged using the BioRad Gel Doc EZ Imager, imaging optimization and analysis were performed using the Gel Doc Imaging Software and Image Lab (Version 6.1.0 build 7), Lane Profiles were exported with ImageJ (Version 1.54k) and plotting of ridgeline plots of intensities was performed in R.

2.4 NanoDrop

DNA and protein concentrations were measured using the Implen NanoPhotometer® N60 in dsDNA mode, using the relevant sample buffer as blank. Samples were measured in technical triplicate, and the mean value is reported.

2.5 Atomic Force Microscopy

Atomic force microscopy was performed on the Bruker Dimension FastScan with the FASTSCAN- D (Bruker) tips (T: 0.145μm, f0:110kHz in H2O, L: 16μm, W: 4μm, k: 0.25N/m) in fluid tapping mode. Scan parameters Tip-velocity, amplitude set-point and drive amplitude were optimized for each imaging session. For 1x1 μm lines x samples /line were set to 512x512 resulting in a pixel size of 1.95 nm, 5x5 μm scans were performed with 1024x1024 with a pixel size of 4.88 nm.
Images were processed in Gwyddion (version 2.63) using the following operations in this order: "Level Data by mean plane subtraction" "align rows with matching" "correct horizontal scars" "Shift minimum Data value to Zero", "false colour theme: gwyddeon.net". False colour scaling was adjusted to yield highest contrast, using "set zero to colour map minimum".

2.6 SDS-PAGE

SDS-PAGE gels were prepared one day prior to use. Stacking gels were cast at 3 percent, resolving gels at 12.5 percent Acrylamide. Recipe for 2 SDS-PAGE gels as follows: Stacking gel (3%): 6.5mL H_2O ,2.5 mL 4X stacking Buffer(0.5M TrizmaBase, pH6.8) , 1.0 mL Acrylamide (30%), 100μL SDS (10%), 100μL Ammonium persulfate (10%), 10μL TEMED. Resolving gel (12.5%): 3.3 mL H_2O
2.5 mL 4X resolving Buffer(1.5M TrizmaBase, pH 8.8), 4.2 mL Acrylamide (30%), 100μL SDS (10%), 30μL Ammonium persulfate (10%), 10μL TEMED.

2.7 ssDNA peptide conjugation, purification and hybridization on DNA Origami

Modified ssDNA (Integrated DNA Technologies, UK) carrying an azide modification at the 5' end, was mixed with an N-terminally DBCO-modified peptide (Peptide Protein Research Ltd, UK) at a 1:1 molar ratio (50 uM final concentration) and incubated at 4 C overnight.
HPLC separation of the overnight azide/DBCO reaction followed (Agilent, UK). HPLC was per- formed using a Clarity 5uM Oligo-RP column (Phenomenex) with a 5-95 % TAE/Acetonitrile gra- dient. The efficiency of the overnight reaction was estimated 90 %. The oligonucleotide/peptide conjugate peak was collected, freeze-dried overnight and resuspended in DNA Origami buffer.

2.8 DNA Nanotructure conjugation

The oligonucleotide/peptide conjugate was mixed with DNA origami carrying ssDNA strands in pre-designed positions complementary to the conjugate sequence (single stranded DNA overhangs) at a 10:1 conjugate:origami molar ratio. The mixture was incubated at 40 °C for 1h and subse- quently cooled down to 20 C with a 1 °C/min rate. For Streptavidin attachment, the biotinylated nanostructures were incubated for 30 min at RT with a 10X excess of Streptavidin to biotin in the sample. The nanostructures were first concentrated by EtOH precipitation as described in section 2.9.

2.9 Purification Methods

Gravity driven size exclusion chromatography (G-SEC) was performed as described in section 1 with the 0.8 CV Micro Bio-Spin™ Chromatography Columns (#7326204) and 5mL CV Pierce™ Disposable Columns (#29922). The two SEC resins used were Sephacryl™ S-300 HR and Cytiva™ superSEC resin.

"Freeze 'N Squeeze" was performed according to the protocol reported in Fu, D et al 2022. Briefly, a 1% agarose gel was prepared as mentioned in 2.3. 50μL of 10 nM Rectangle, together with 10μL Loading Dye was loaded into extended loading pockets (created by excision), then run for 30 minutes. The bands of interest were excised and loaded into the BioRad Freeze 'N Squeeze™ DNA Gel Extraction Spin Columns and frozen at −20 °C as described in [7] then centrifuged to extract DNA nanostructures.

The sucrose elution method was performed according to the protocol reported in [8]. A 10mL 4% agarose gel without DNA stain was prepared, poured and left to set, then 40mL of 1% agarose gel, prepared as described in 2.3 was poured on top. An elution pocket below the bands of interest was excised, the electrophoresis tank was emptied by 20% of running buffer volume to make sure no buffer entered the excised pocket, and the gel was placed back into the chamber and the running buffer was refilled. The pocket was then filled with 30% sucrose solution containing 5mM $MgCl_2$.

Ethanol-mediated precipitation was performed according to the protocol presented in [9]. The folded nanostructures were adjusted to 30% EtOH and centrifuged for 1h at 6,000 rpm. The supernatant was removed, and the sample was left to dry for 10 min,

ensuring that all EtOH has evaporated. The desired volume of Origami Buffer was added to the sample, and it was resuspended by pipetting. The sample was incubated overnight at room temperature before use.

Ultrafiltration was performed using Amicon® Ultra Centrifugal Filters, 100 kDa MWCO. The filter was prewetted with 500 μL buffer. Sample was then added, and the volume was adjusted to 450 μL with origami buffer. The sample was washed three times, by centrifugation for 3 minutes at 1,000 rcf. The remaining solution was then recovered and volume measured by pipetting.

FPLC was performed with ÄKTA pure™ using the Superose 6 5-150 increase GL SEC column. The fraction collector was set to collect 0.1 mL fractions and the flow rate was set to 0.5mL/min ensuring no overpressure. The elution buffer was 1X Origami Buffer vacuum filtered through a 0.22μm filter. Column was equilibrated with 0.5 CV Elution Buffer.

2.10 Function Fitting NanoDrop Data

Purity and resolution of the run can be calculated by fitting the peaks with 2 or more functions as shown in equation 1. As the peaks are not gaussian we use the probability density function of a log normal distribution. Because of the two very different sizes (DNA Origami vs. Staples) two distributions are expected the sum of the two distributions should be able to describe the measured distributions.

$$y = \frac{a_1}{x \cdot \sigma_{1\sqrt{2\pi}}} \cdot \exp\left(-\frac{(\log(x) - \mu_1)^2}{2\sigma_1^2}\right) + \frac{a_2}{x \cdot \sigma_{2\sqrt{2\pi}}} \cdot \exp\left(-\frac{(\log(x) - \mu_2)^2}{2\sigma_2^2}\right)$$

Where y is the concentration of an elution fraction as measured by NanoDrop, x is the elution volume, μ is the logarithmic mean of the distribution in x (elution volume), σ is the standard deviation of the logarithm of the elution volume and $\alpha 1$ and $\alpha 2$ are positive scaling constants.
The resolution is calculated by using the resolution definition for close peaks taking the FWHM approach as shown in equation 2. R_S is the Resolution, t_R is the x coordinate of the peak n, $w_{1/2}$ is the half peak maximum width.

$$R_s = \frac{2 \cdot (t_{R2} - t_{R1})}{w^1_{\frac{1}{2}} + w^2_{\frac{1}{2}}}$$

We used R for fitting, plotting and calculating purity and resolution. The results of the purification of different nanostructures are shown in Figure S4.

Conceptually we fit the function by visually guessing at the start parameters, altering the param- eters of the individual functions till the peak height, x coordinate and width are roughly what is expected use these parameters, repeat for the second peak. If the parameters are too far off the fit will fail to find parameters; however, this only needs to be done once for the initial guess of a certain CV and loading volume as the peak width and elution volume will not change much across runs and the fitting is robust enough for small changes. Purity is calculated by dividing the fraction area / fraction by total area under the curve of the fitted combined function, this is done numerically, not analytically.

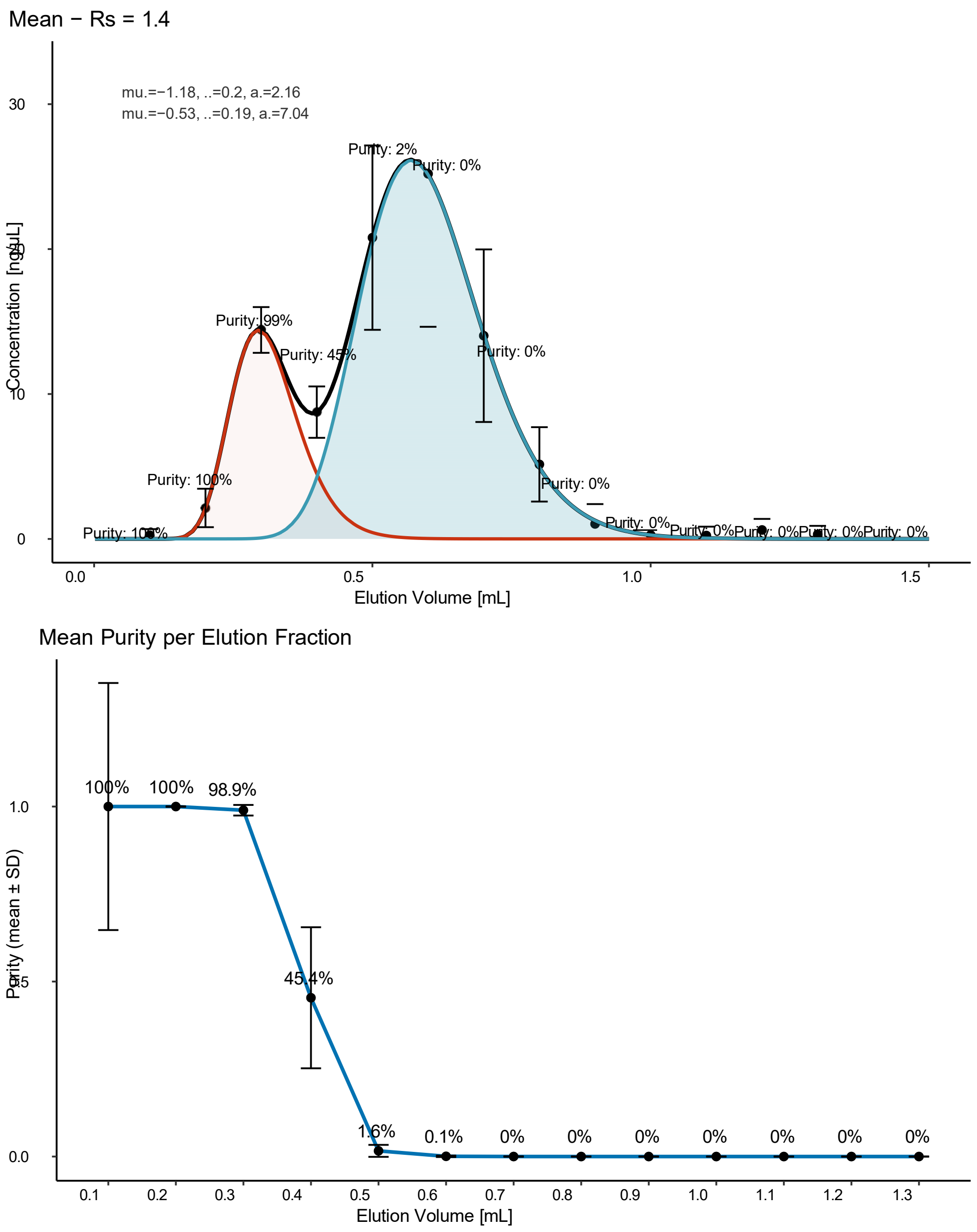

Figure S4: Plots showing the fit of the mean as well as the purity by fraction.

3 Additional Data

3.1 Cytiva S-300 vs SuperSEC performance in 0.8 mL CV

We set out to compare the performance of purification using the Sephacryl S-300 resin to that of the SuperSEC resin in gravity driven SEC spin columns of 0.8 mL CV. We observed that staple separation from formed nanostructures is visibly more efficient with SuperSEC resin than S-300 in gravity-driven SEC (Figure S5). Nanodrop concentration measurements show no clear peak separation for the S300 sample, and the agarose gel shows remaining staple strands in fraction 3 and fraction 4 (300 & 400 μL elution).

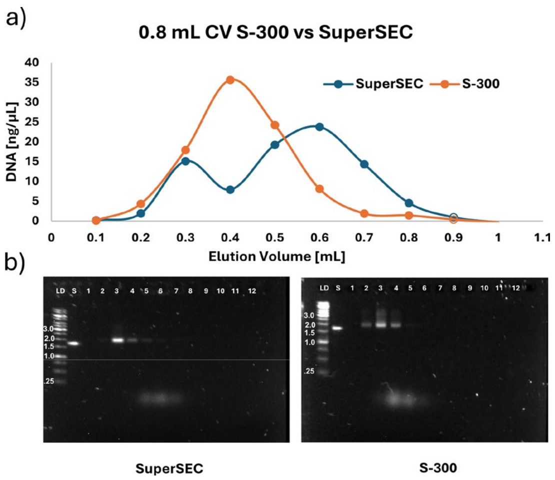

Figure S5: Comparison of S-300 vs SuperSEC size exclusion resin in Gravity-SEC applications **a)** Concentrations measured by nanodrop for 50μL 10nM (2.5 μg) R run for both resins, interpolation as visual aid. **b)** AGE images of the gels run for the samples measured in **a.** On the left is the gel for SuperSEC and on the right the gel for S-300.

3.2 Loading volumes for 0.8 mL CV Column

We assessed the performance of the 0.8 mL CV G-SEC on 25, 50 and 100 μL loading volumes (LV). 50 μL showed the best separation at maximal loading volume. The 50μL LV delivers the best performance with highest loading capacity as shown in the comparison in Figure S6.

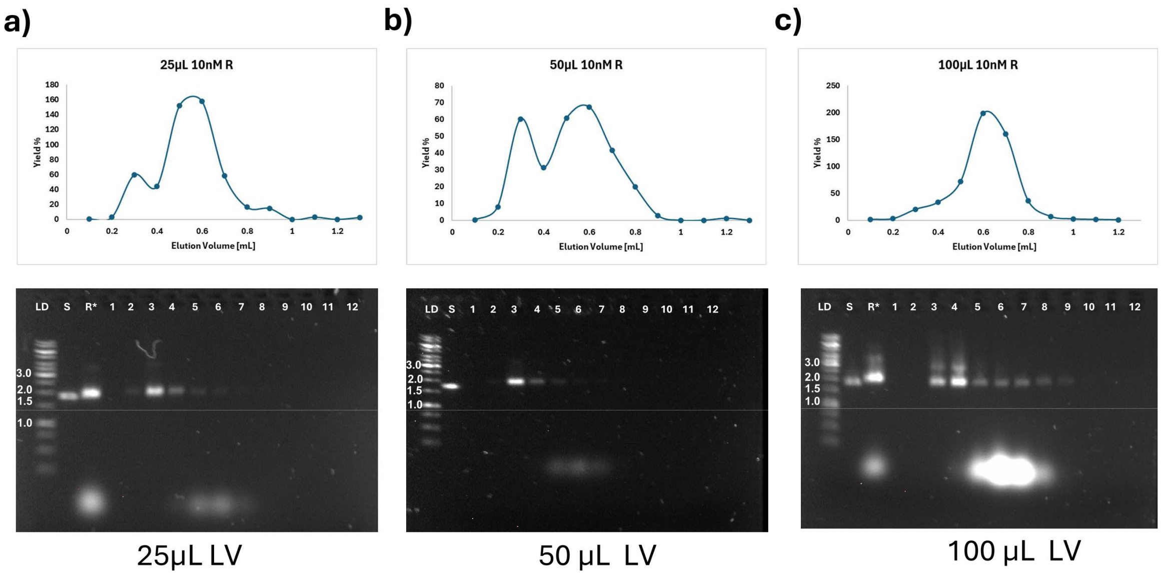

Figure S6: NanoDrop DNA concentration measurements in % yield of different sample loading volumes [a) 25μL, b) 50μL and c) 100μL] scatter plots vs the elution volume, interpolated as visual aid [upper] and representative AGE images of the fractions [lower].

3.3 Column Volumes and Protein Seperation

We tested three column volumes for protein separation using 10 nM of Rectangle nanostructures: 0.8 mL (50μL loading volume, 100μL fractions), 2.5 mL (100μL Loading Volume, 125μL fractions) and 5mL (100μL loading volume, 250μL fractions). The results of the comparison are shown in Figure S7. Examples of the columns are shown in Figure S7a. Separation and elution peak breadth increases with increasing CV as seen in Figure S7b while dilution increases with rising CV. Streptavidin is easily separated from both the Rectangle and the Rectangle-streptavidin conjugates as shown in Figure S7c for the example of 0.8 mL CV and similarly higher CVs separate the nanostructures from Streptavidin as shown in Figure S7d. From this we conclude that using higher CVs than 0.8 mL is counterproductive for small volume purifications of about 50 μL although higher loading volumes such as shown in Figure S6c might result in reduced separation.

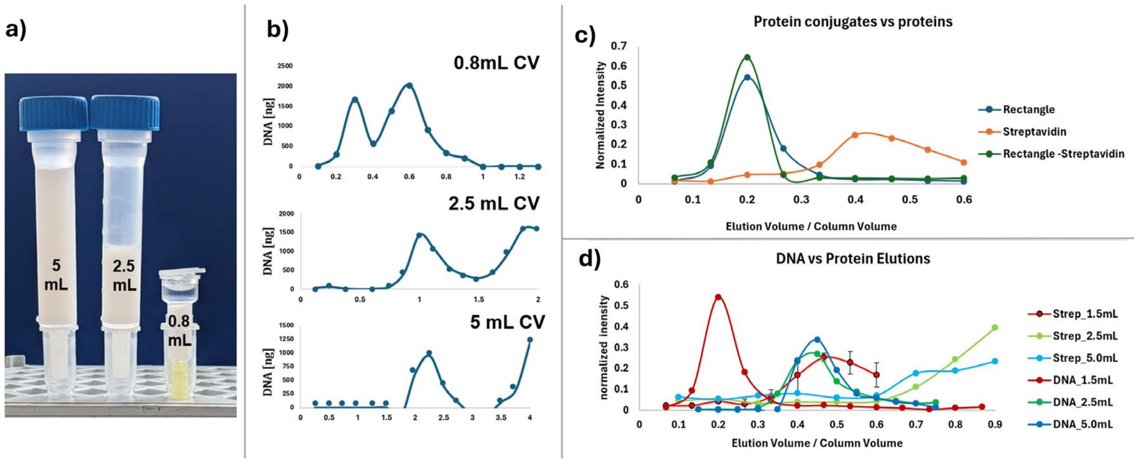

Figure S7: **a)** Picture comparing 5 mL, 2.5 mL and 0.8mL CV spin columns on 0.8mL eppendorf tubes. **b)** Total DNA weight estimated by nanodrop concentration measurements for purifications from the 5 mL, 2.5 mL and 0.8 mL CV versus the elution volume. **c)** Shows the normalized intensity extracted from gel images of agarose gel electrophoresis and SDS- PAGE of the Rectangle Nanostructure (AGE), the Rectangle conjugated to Streptavidin (AGE) and streptavidin (SDS-PAGE) versus the elution volume divided by the column volume. **d)** Shows the normalized intensity of DNA Nanostructures in AGE and Streptavidin in SDS-PAGE vs the Elution Volume divided by the column volume.

Complimentary to Figure S7 we show the comparison of NanoDrop concentration measurements for the fractions of the unconjugated, Streptavidin-conjugated rectangle and the TBP-conjugated Triangle below in Figure S8. Notably both the Triangle and Rectangle were EtOH precipitated before conjugation, 500 μL 10.6 nM 25 μg nanostructure + 217μL EtOH, resuspended in 100 μL Origami buffer. 30 μL of the resuspended nanostructures were then conjugated with streptavidin or TBP and purified on the 0.8 mL CV SuperSEC columns with PBS supplemented with 7 mM MgCl2 as described in section 2.1. The difference between the Rectangle and Triangle in the main elution peak is minimal, yet the nanodrop clearly shows the ssDNA-TPB being separated from the Triangle nanostructure (see also Figure 3.a).

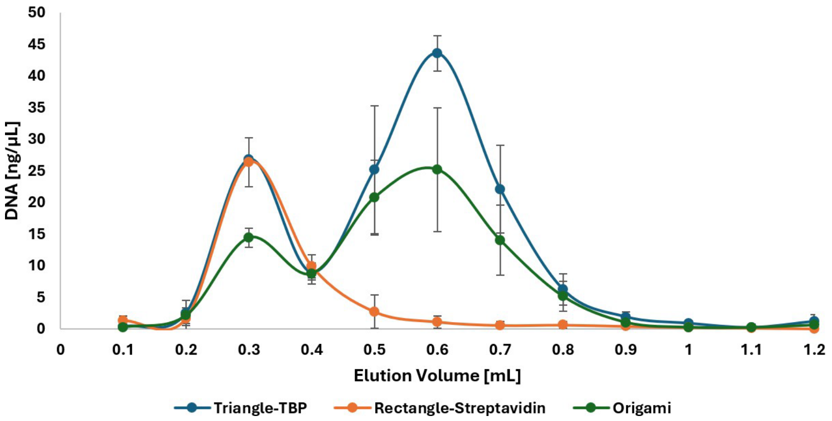

Figure S8: Comparison of the concentration measurements of Triangle-TBP conjugate (N=3), Rectangle-Streptavidin conjugate (N=3) and the mean (N=7) of all nanostructures as shown individually in S10 gravity purified on the 0.8 mL CV SuperSEC column.

3.4 Nanostructure integrity

To assess the effect of purification on the structural integrity of the nanostructures, we counted the intact vs non-intact structures before and after purification. This was performed with 5WF, frame, triangle, and rectangle. The annotated images to the data in S2 are shown in the addendum. Note that the AFM tip can destroy nanostructures which means that areas scanned for longer will have increasing numbers of defective nanostructures. The counts were performed with clickmaster2000 : https://www.thregr.org/wavexx/software/clickmaster2000/

Nanostructure	Intact	Broken	Intact (%)	Broken (%)
5WF	1212	753	61	38
5WF F3	311	245	56	44
Frame	61	13	82	17
Frame F3	47	11	81	18
Triangle	75	17	81	18
Triangle F3	65	17	79	20
Rectangle	1937	612	75	24
Rectangle F3	754	84	89	10

Table S2: Counts and percentages of Nanostructures divided between intact and defective.

3.5 Purification of different Nanostructures

As previously shown in main paper Fig 2.d we tested the 0.8 mL CV SuperSEC columns for several nanostructures, the unpurified vs purified structures are shown with their corresponding gels below in Figure S9. The concentration measurements of the samples ran on the gels in Figure S9 are shown in Figure S10.

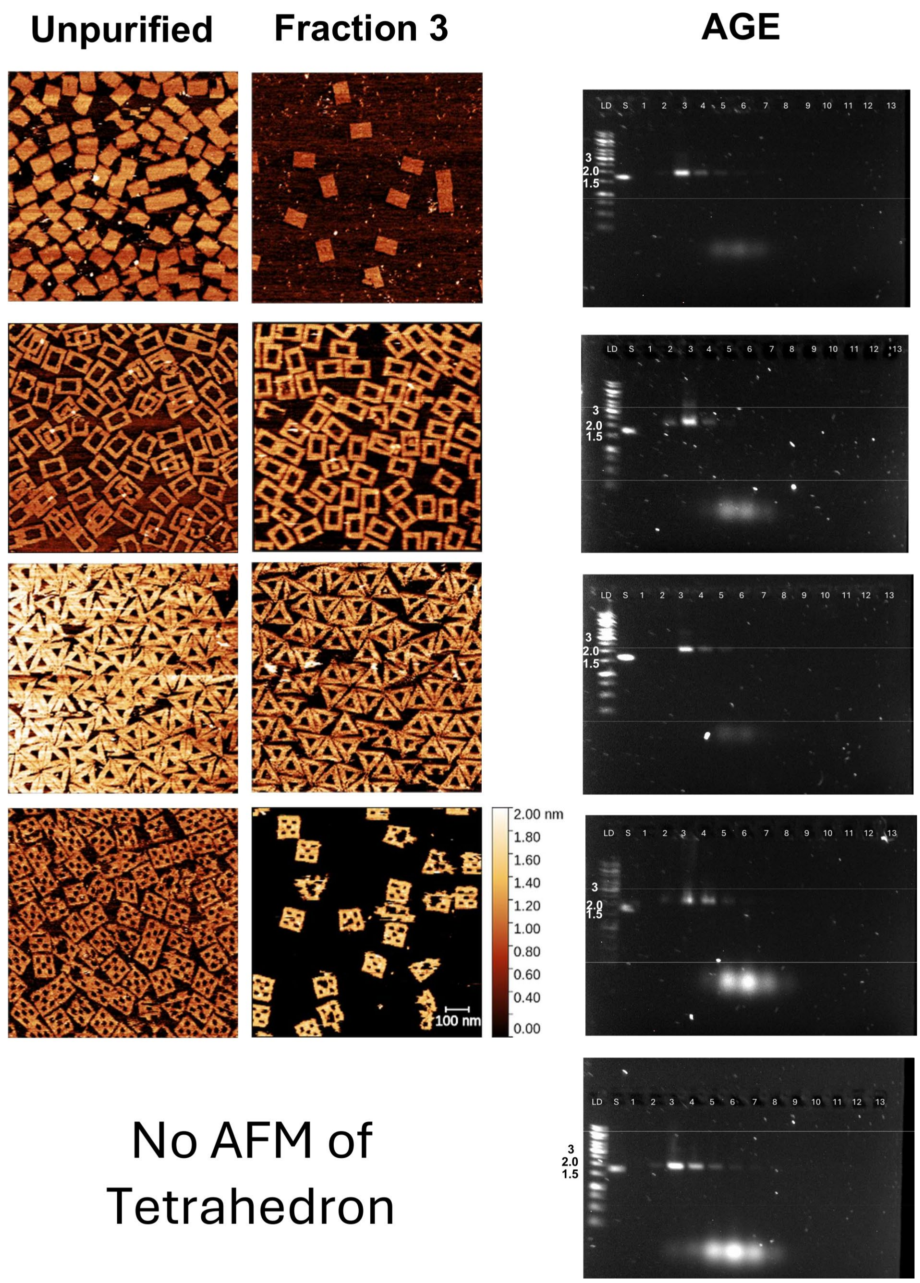

Figure S9: AFM of unpurified vs purified nanostructures for rectangle, frame, triangle, 5-well frame and tetrahedron* and gels for their respective purifications. 50μL of 10nM folded nanostructure were purified by a 0.8mL CV SuperSEC column with 100μL elution fractions.

The AFM of the tetrahedron structure is missing as the structure's geometry and lack of large surface area for mica interaction does not lend itself to AFM imaging. Characterization of the tetrahedron was performed using Transmission Electron Microscopy (Figure S9.b).

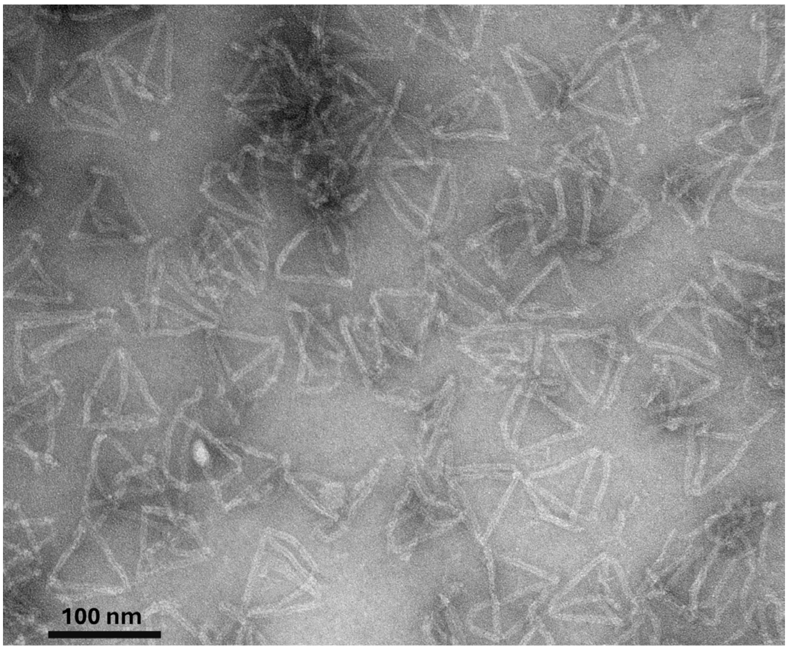

Figure S9.b: TEM of DNA 3D Tetrahedra, corresponding to the tetrahedron sample in Figure S9.

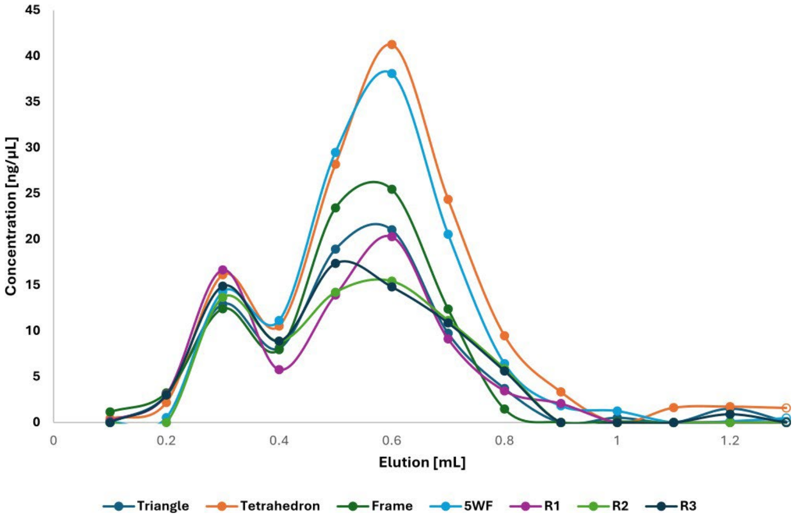

Figure S10: Shows the concentration measurements vs elution volume for 50 μL 10nM LV on the 0.8 mL CV SuperSEC with 100μL fractions for each of the Nanostructures shown in Figure S9. Three replicates are included for the Rectangle nanostructure as R1, R2 and R3. The interpolation serves as a visual guide only.

3.6 Reusability

To test the reusability of the columns by clean-in-place methods common to SEC columns for FLPC systems, we washed the 0.8 mL CV SuperSEC columns with 1 CV elution buffer after each purification of DNA Nanostructures. We used columns up to 10 times before returning the SEC resin to a falcon tube for collecting the resin from several columns to be washed more thoroughly by NaOH and stored in 30% EtOH for reuse. Below in Figure S11 we show the first (Rectangle) and tenth (Triangle) purification of DNA Origami on a 0.8 mL CV column.

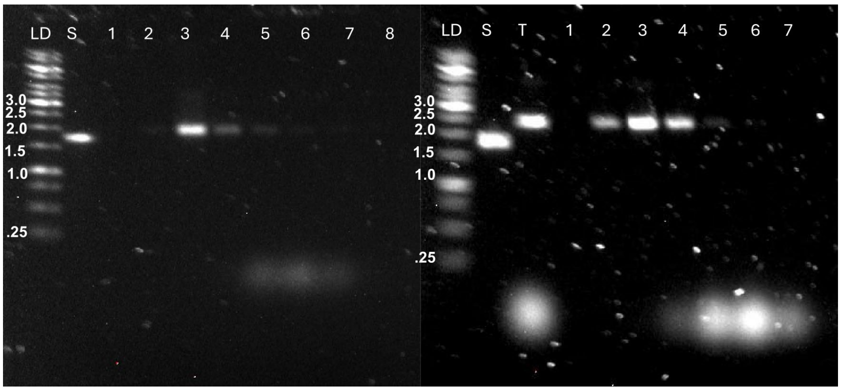

Figure S11: Reusability: For a 0.8mL CV SuperSEC column first (left - Rectangle) and tenth (right -Triangle) purification are shown.

3.7 Purification Method Comparison

We tested several methods for purifying DNA Origami, the two common Gel extraction techniques: Sucrose Elution and FreezeNsqueeze, precipitation via Ethanol and the Ultrafiltration by Molecular weight cut off (MWCO). The methods were performed as described in section 2.9.
The Sucrose elution method can work quite well for functionalized nanostructures S12a however the method is highly user dependent and difficult to repeat, new users will struggle getting it right the first few times. It is even harder when one does not have a way to standardize the cut out process for the elution pocket. Over 7 repeats the sucrose elution method yielded 38+-22% of input.
Freeze and squeeze S12a also relies on cutting out the minimum amount to extract the whole gel segment containing the desired nanostructure. This method recovered 38+-47% on input nanostructures. In several cases no recovery was possible, making this method unreliable at best. Both gel extraction methods are clearly not scalable and both contaminate the DNA irreversibly with a DNA stain as well as presenting low recovery and significant reliability issues. MWCO (100kDa) filtering had varying results yielding 65+-16%. In our earlier tests loading 25 μL of a 10nM 5WF solution did not recover any DNA nanostructures, in tests with Streptavidin we saw no separation as the Streptavidin still interfered with AFM imaging.

The purification by Ethanol was the method with the highest variability, from recovering almost nothing S12b EtOH(1) to recovering 200% S12b EtOH(4) final mean yield of 79+-74% of 2.5μg Origami in 50μL (10nM), all yields are uncorrected for purity. Recovering DNA Origami from anything less than a 50μL (2.5μg) starting volume / mass is very hard as there is seldomly a visible pellet. The method provides highly varying degrees of purity as can be seen in S12b as well as having an often unreliable recovery. However, the reliability increases with scale: using the method on 500μL Rectangle DNA Origami recovered a pellet in every case. This is why we recommend this method as a concentration step rather than a purification step, with higher amounts of starting material and a tolerance for impurities.

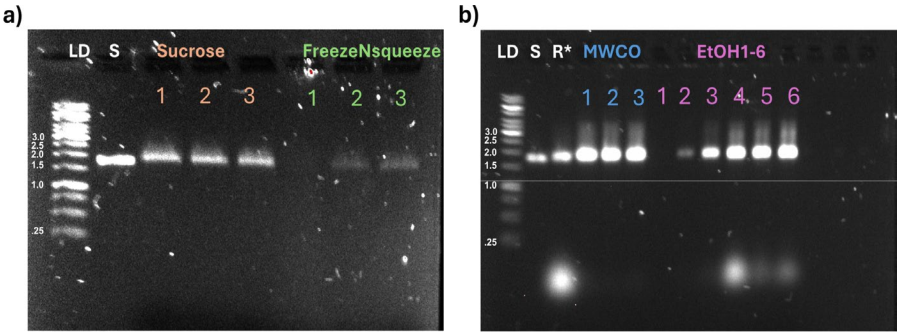

Figure S12: Four alternative purification methods. a) Eluting into Sucrose from an AGE method and the FreezeNsqueeze method both in triplicate. b) Molecular weight cut off filtration by centrifugation in triplicate as well as a sextuplicate of the precipitation by EtOH. Scaffold DNA is marked by "S" and unpurified Rectangle is marked by "R*".

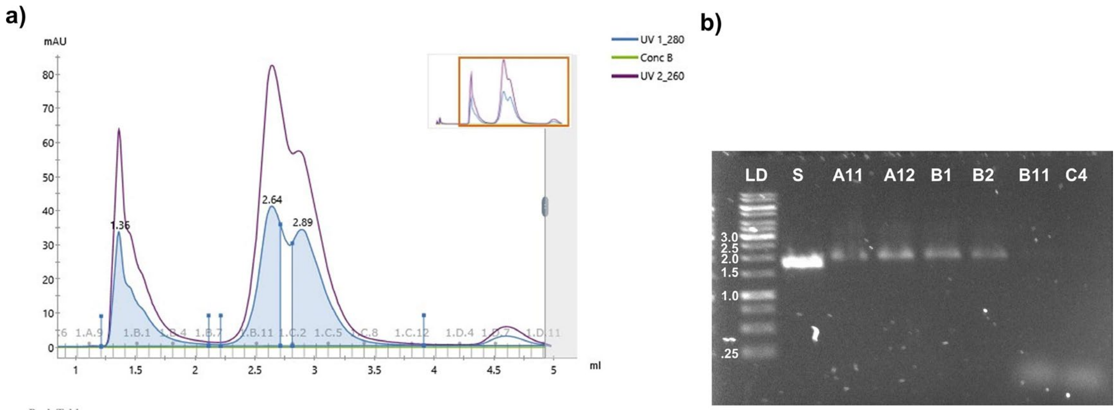

Peak Table

Peak	Retention (ml)	Area (ml*mAU)	Area %	Ext coeff. (mg $^{-1}$ ml cm $^{-1}$)	Fraction(s)	Volume (ml)
Peak A	1.358	6.714	27.18		1.A.10 - 1.B.6	0.900
Peak B	2.637	7.972	32.27		1.B.8 - 1.B.12	0.500
Peak C	2.892	10.02	40.55		1.C.2 - 1.C.12	1.100

Figure S13: Shows a representative purification run of 100μL 10 nM freshly folded Rectangle purified on the Äkta-Pure with a 5x150 Superose 6 SEC column, the Origami containing fractions represented by peak A yield 36% with fraction A12 at 1.1mL elution volume containing the highest concentration with 4.5 ng/μL representing a 12X dilution at 9% recovery. b) An AGE image of the first peak from A11 to B2 and the two following peaks B11 and C4 are shown in comparison to the scaffold, 20μL is loaded per fraction, 5 μL Ladder and 5μL 10nM ssDNA scaffold.

3.7.1 EtOH precipitation - 25μg DNA Origami

In Table S3 NanoDrop measurements and yields of EtOH precipitations of larger amounts of DNA Origami are shown. The previous data on EtOH precipitation shown in S12 were obtained from 50μL of 10 nM Rectangle (2.5μg). In the experiment summarized in Table S3, 500μL of 10 nM DNA Origami (25μg) were precipitated in 30% EtOH for the Rectangle and 25% for the Triangle, following [9]. The precipitates were resuspended in 100μL Origami Buffer. The 100μL samples were then split into three 30μL samples to provide technical triplicates for the Rectangle–Streptavidin and Triangle–TBP conjugation reactions.

Overall, the yield for EtOH precipitation of 500μL of DNA Origami (25μg) was 58.44±41.04μg or 233.8±164.2%.

Sample	ND1[ng/μL]	ND2[ng/μL]	ND3[ng/μL]	Mean[ng/μL]	SD[ng]	Yield [μg]	Yield [%]
R1 (1.9.24) 1	168.9	168.25	168.1	168.42	0.43	16.84	67.3
5WF1(4.9.24)	305.9	322.0	-	313.95	-	31.39	125.6
5WF2(4.9.24)	1260.1	1267.2	-	1263.65	-	126.37	505.5
T1(4.9.24)	464.5	-	-	464.5	-	46.45	185.8
T2(4.9.24)	260.85	265.2	-	263.03	-	26.30	105.2
T32(14.01.25)	603.35	603.05	602.5	602.99	0.43	60.29	241.2
T4 (20.01.25)	1010.3	1012.4	1020.1	1014.26	5.16	101.43	405.7

Table S3: NanoDrop measurements of precipitation of 500 μL 10 nM DNA Origami with 25-30% EtOH resuspended in 100 μL of Origami Buffer. R and 5WF were precipitated with 30% EtOH and Triangle with 25%.

3.8 Method comparison Literature

Although there are many papers reporting a variety of different methods for purifying DNA Origami few report the achieved yield and purity, the following review lists the majority([10]). Often the exact methods are reported very briefly so that no greater insight is possible. The table below does not represent an exhaustive list but an overview of important method papers and papers that have previously compared purification methods for DNA Origami and Protein conjugated DNA Origami. The data from this table informed Figure 5 in the main paper.

Paper	Method	Yield	Purity	Time
Facile and Scalable Preparation of Pure and Dense DNA Origami Solutions [11]	PEG (7.5%)	∼96%	>96%	0.5 h + 20 h
	MWCO	53%	92%	>0.5 h
	AGE	∼25%	>99%	>2 h
Purification of Functionalized DNA Origami Nanostructures [1]	MWCO	85%	95%	0.5–1 h + OV
	Gel Extr.	15–30%	>80%	4.5–5 h + gel prep
	Gel Filt.	55–70%	>85%	0.5 h + conc
	Glycerol Ultr.Cent.	25–35%	30–>98%	>3 h
	PEG (3.5%)	45-70%	85-95%	–
	Mag. Bead	30–70%	>95%	OV + >7 h
	FPLC (Sup6)	55–65%	>99%	–
A Robust and Efficient Method to Purify DNA-Scaffolded Nanostructures by Gravity-Driven Size Exclusion Chromatography [12]	G-SEC	50-70%	>99%	0.4-1h
Automated Purification of DNA Origami with SPRI Beads[13]	SPRI (0.8X)	49%	89%	0.5–1 h
	FPLC (S400HR)	25%	>99%	–
	MWCO	49%	31-94%	–
	PEG (7.5%)	∼20%	97%	–
	Phase.Sep	∼25%	71%	–
	EtOH	∼100%	36%	–
How we make DNA Origami [14]	AGE	10–35%	–	4 h
	MWCO	60–99%	–	0.5–3 h
	FPLC-SEC	70–90%	–	3h
	AGE	8-38%	-	2-3h
	Ultracentrifugation	up to 98%	–	0.25–1 h
	Ultra DGC	40–80%	–	3–4 h
	PEG	–	–	1h
This work	AGE (FQ)		>95%	3h
	AGE(Sucrose)	38±22%	>95%	3-4h
	EtOH	79±74%	50-90%	1h + OV
	MWCO(100kDa)	65±15%	>95%	0.5h
	Gel. Filt	35%		0.25h
	FPLC-SEC	36%	>99%	3-4h
	G-SEC	92%	90-99%	0.1-0.5h

Table S4: Overview of DNA origami purification methods with reported yield, purity, and pro- cessing time. Purities for the SPRI paper were calculated from Figure 3, the table of extracted purity is shown below in S5.[13]Ranges are given when reported. FPLC is always associated with high dilution. Precipitation techniques often show aggregates. Notes and abbreviations: OV -> Overnight step, conc -> concentration.

Lane	Sample	Purity
2	Uncleaned 4FST tile	0.226
3	0.8X SPRI	0.887
4	S-400 HR spin column	0.968
5	100 kDa MWCO-1	0.939
6	100 kDa MWCO-2	0.311
7	PEG precipitation (PEG)	0.970
8	Phase separation (P.S.)	0.711
9	Ethanol precipitation	0.364
10	Size-exclusion column (SEC)	0.99

Table S5: Data extracted from SPRI purification paper from Figure 3.[13] Purity of DNA origami samples calculated from lane profile peak integration. Purity is defined as Origami / (Origami + Staples).

4 Reproducibility in an Independent Laboratory

To test the reproducibility of our method we provided the SuperSEC resin and 0.8 mL columns to the Single-molecule Sensing Group at the Max-Planck Institute for Medical Research in Heidelberg. The purification protocol was used in the laboratory with a two-layer DNA Origami rectangle structure, both plain, and functionalized with zinc-porphyrin as well as aptamers. The three nanostructures were plain (DR) and DR + Zinc Porphyrins (DR-ZnP) and lastly DR-ZnP with aptamers (DR-ZnP-Apt).

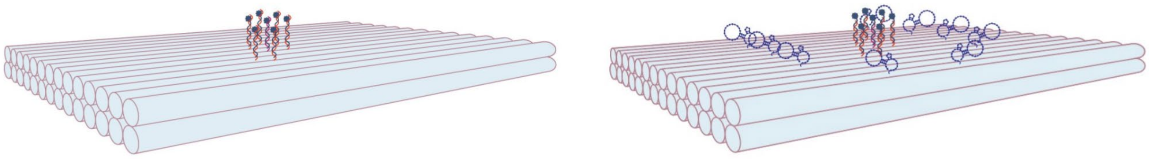

Figure S14: Showing the mockup of nanostructures DR + Zinc Porphyrins (DR-ZnP) and DR-ZnP with aptamers (DR-ZnP-Apt). The plain nanostructure DR simply lacks the ZnP ssDNA functionalization.

4.1 Gels and AFM imaging

To assess the elution behavior and correct folding of the DNA Origami, AFM and agarose electrophoresis were performed. As shown in S15 and S16 the DNA Origami was recovered intact and is successfully functionalized with ssDNA carrying Zn-Porphyrins. Gel electrophoresis shows very similar elution behavior compared to the DNA Origami tested in Cambridge (S15). DNA Origami were either purified directly after folding (DR) or first filter-purified via a 100kDa MWCO Amicon filter then incubated with 350 nM of Zinc-porphyrin ssDNA complimentary to 7 overhangs on the nanostructure surface (DR+ZnP). Lastly a sample of DR was folded with aptamers as overhangs then conjugated to the ZnP-ssDNA post MWCO filtration (DR+ZnP+Apt). The three types of DNA Origami elute in Fraction 3 (note the MPI results are zero-indexed with the first fraction being loading volume void).

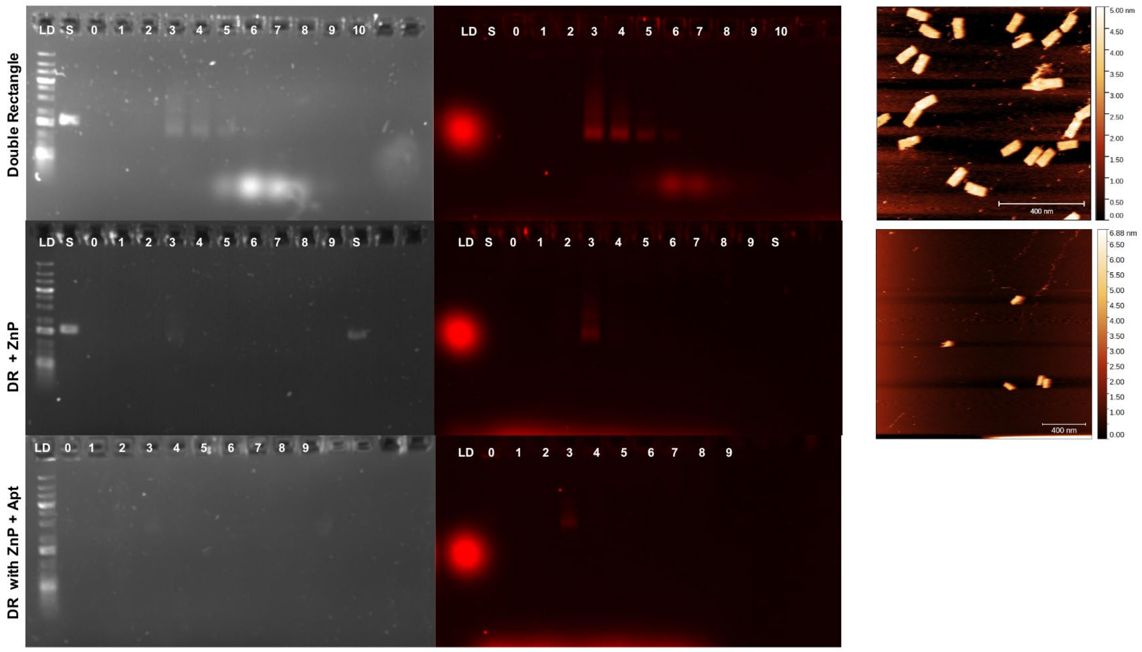

Figure S15: Data from reproduction of method in the Glembockyte Lab. Gels shown for the double rectangle, the DR+Aptamer and DR+Zinc Porphyrin. AFM in height mode shown for DR, in error mode for DR+. Fraction Elution is 0 indexed as the loading EV was collected separately as F0.

Although the AFM quality could be improved in the future, it is nonetheless possible to confirm the functionalization of the DNA Origami with the ZnP-ssDNA linkers. These are present as a 0.5 nm bump in the middle of the DR Origami as shown below in Figure S16 and in the height profile in S17 . The error trace signal shown in Black and white in S15 and in S16 gives a higher contrast and lets us see the ZnP attachment more clearly. The ZnP attachment is confirmed in S16.

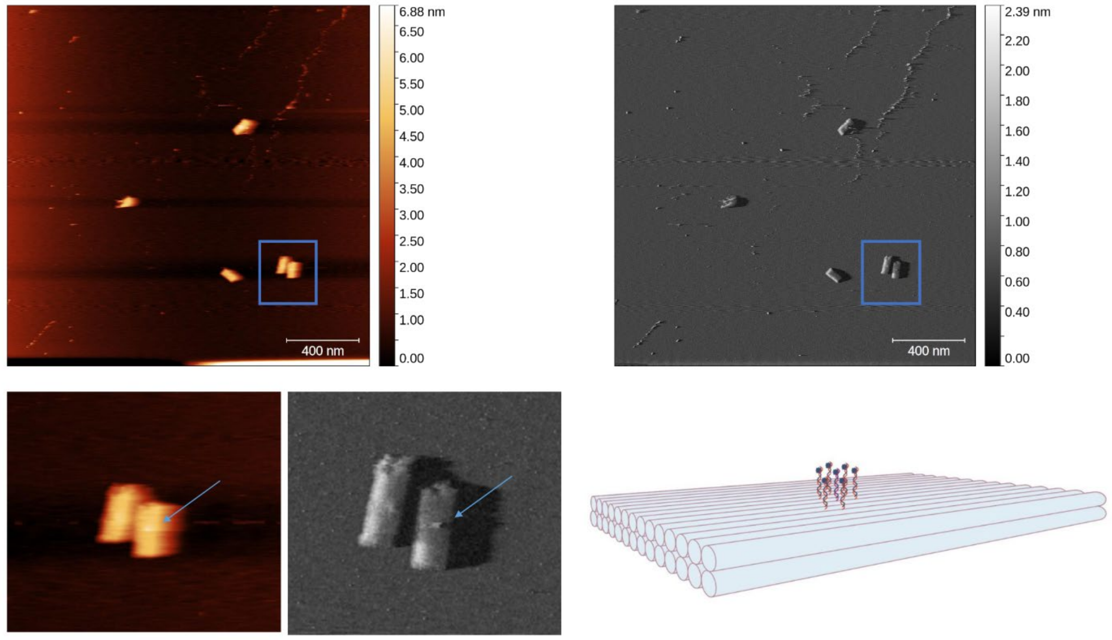

Figure S16: AFM images of the DR-ZnP fraction 3, Height trace shown in Gwyddeon.net colour scheme and the error trace shown in greyscale. Enlarged cutouts of the AFM images are shown on the lower left, their cut-out regions marked on the larger images. Arrows point towards the ZnP attachment site.

The attachment of the ZnP-ssDNA can further be verified by the 0.5nm bump seen in the height profiles of the two DR Origamis shown in the screenshot of Gwyddion measurements of the AFM.

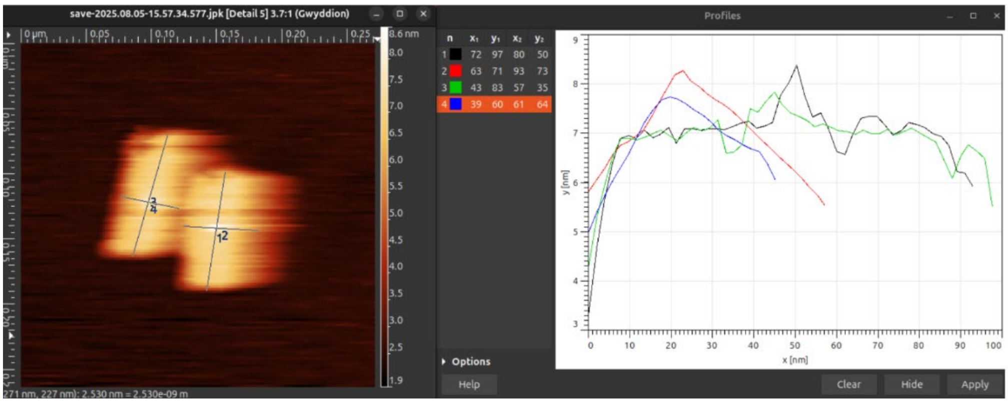

Figure S17: Height Profiles of two DR-ZnP DNA origami showing the ZnP ssDNA attachments in the center of the structure.

4.2 Yields

Table S6: NanoDrop concentration measurements of the eluted fractions.

Fraction	F3	F4	F5	F6	F7	F8	F9
DR [ng/μL]	6.7	4.2	12.3	51.7	35.4	12.5	2.4
DR +ZnP [ng/μL]	7.55	3.8	2	2	-	-	-
DR +ZnP+Apt [ng/μL]	4.2	1.8	2	1.8	-	-	-

Table S7: Yields calculated for the single replicate 0.8 mL CV G-SEC (SuperSEC) columns with the purification buffer described in S10

	Load [ng]	Yield [ng]	Yield [%]
DR	2620	1090	41.6
DR +ZnP	1750	1135	64.8
DR +ZnP+Apt	1750	600	34.3

4.3 Methods

4.3.1 DNA Origami Folding

The double rectangle structures were folded at 10nM using the p8064 m13 scaffold in the buffer shown below in S8. Folding was conducted according to the protocol in S9. Buffer used in the G-SEC purifications as shown in S10. The design of the structure was previously reported in Scheckenbach et al 2025. [15]

Table S8: Folding buffer composition

Component	Concentration
$MgCl_2$	16 mM
Tris	10 mM
EDTA	1 mM
NaCl	5 mM
Scaffold	10 nM
Staples	100nM

Table S9: Folding program parameters

Parameter	Value
Lid temperature	100 °C
Reaction volume	100μL
Step 1	70 °C, 5 min
Step 2	53 °C $\rightarrow$ 37 °C, 1 °C/h
Step 3	25 °C, 10 min
Step 4	4 °C, hold
Total time	~17 h

Table S10: Purification buffer composition

Component	Concentration
Tris	5 mM
EDTA	1 mM
$MgCl_2$	5 mM
NaCl	300 mM

4.3.2 AFM imaging

AFM imaging was performed with the Bruker NanoWizard Ultraspeed2 with the FASTSCAN-D tips [T:0.145μm, f_0=110kHz in water, L = 16μm, W = 4μm, k = 0.25 N/m] in water on a freshly cleaved mica sheet.

4.3.3 Gel imaging

Yield: 1. 50µL 10nM +100nM Staples(8064) (5.42MDa) 2. 35ng/µL *50µL [6.4nM] start DR+ 350nM Staple 3. 35ng/µL *50µL [6.4nM] start DR+ 350nM Staple

5 Quantification of streptavidin attachment

For the quantification of streptavidin binding only undamaged well-formed nanostructures were considered. The number of streptavidin molecules per nanostructure were counted and the resulting distribution is shown below:

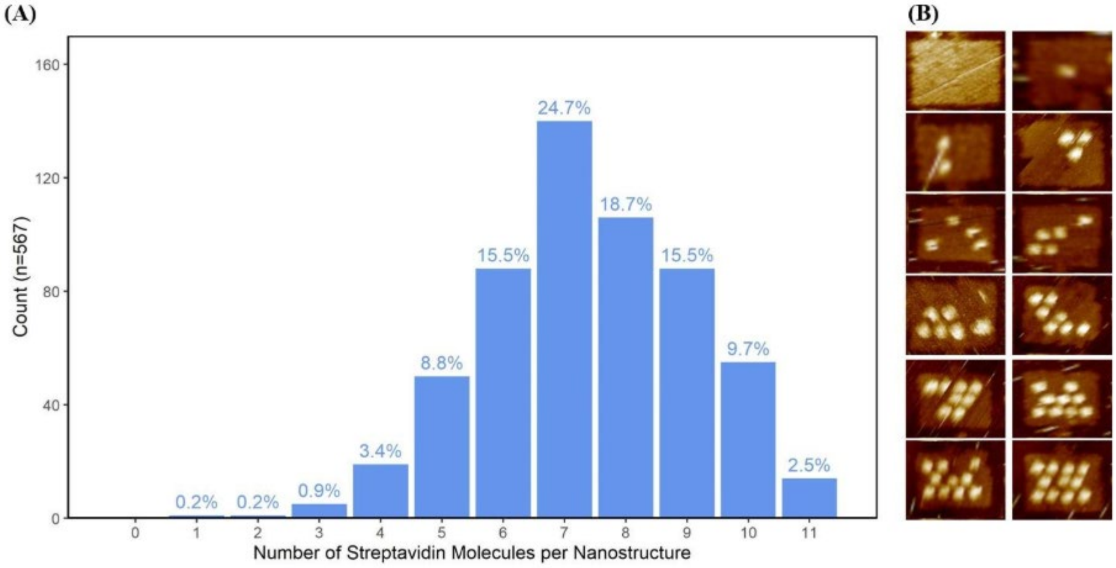

Figure S18: Quantification of the streptavidin binding efficacy. (A) The number of streptavidin molecules bound to biotin nanostructures follows a negatively skewed distribution with its peak at 7 streptavidins per nanostructure (n=567). Created with RStudio. (B) Individual nanostructures can bind different numbers of streptavidin molecules.

6 Code Availabilty

Code is available at the following link: https://github.com/izarscharf/FittingNanoDrop/

7 Bibliography

1. Shaw, A., Benson, E. & Högberg, B. Purification of Functionalized DNA Origami Nanostructures. *ACS Nano* **9,** 4968–4975. issn: 1936-0851 (May 2015).
2. Kielar, C. *et al.* On the Stability of DNA Origami Nanostructures in Low-Magnesium Buffers. *Angewandte Chemie International Edition* **57,** 9470–9474. issn: 1521-3773. /doi/pdf/10.1002/anie.201802890%20https://onlinelibrary.wiley.com/doi/abs/10.1002/ani.201802890%20https://onlinelibrary.wiley.com/doi/10.1002/anie.201802890(July 2018).
3. TBE buffer. *Cold Spring Harbor Protocols* **2006,** pdb.rec8458. issn: 1940-3402 (June 2006).
4. TAE Buffer (50 ¡i¿×¡/i¿). *Cold Spring Harbor Protocols* **2018,** pdb.rec098756. issn: 1940- 3402 (Mar. 2018).
5. TE Buffer (5×). *Cold Spring Harbor Protocols* **2016,** pdb.rec092320. issn: 1940-3402 (May 2016).
6. Castro, C. E. *et al.* A primer to scaffolded DNA origami. *Nature Methods* **8,** 221–229. issn: 1548-7091 (Mar. 2011).
7. Fu, D. *et al.* Automated design of 3D DNA origami with non-rasterized 2D curvature. *Science Advances* **8.** issn: 23752548.

https://www.science.org/doi/10.1126/sciadv.ade4455
(Dec. 2022).

8. Bellot, G., McClintock, M. A., Lin, C. & Shih, W. M. Recovery of intact DNA nanostructures after agarose gel–based separation. *Nature Methods 2011 8:3* **8,** 192–194. issn: 1548-7105. https://www.nature.com/articles/nmeth0311-192 (Feb. 2011).

9. Lei, Y., Mei, Z., Chen, Y., Deng, N. & Li, Y. Facile Purification and Concentration of DNA Origami Structures by Ethanol Precipitation. *ChemNanoMat* **8,** e202200161. issn: 2199-692X. https:// onlinelibrary. wiley. com/ doi/ full/ 10 . 1002 / cnma. 202200161
(July 2022).

10. Knappe, G. A., Wamhoff, E.-C. & Bathe, M. Functionalizing DNA origami to investigate and interact with biological systems. *Nature Reviews Materials* **8,** 123–138. issn: 2058-8437 (Dec. 2022).

11. Stahl, E., Martin, T. G., Praetorius, F. & Dietz, H. Facile and Scalable Preparation of Pure and Dense DNA Origami Solutions. *Angewandte Chemie (International Ed. in English)* **53,** 12735. issn: 15213773. (Nov. 2014).

12. Ebrahimimojarad, A. *et al.* A Robust and Efficient Method to Purify DNA-Scaffolded Nanos- tructures by Gravity-Driven Size Exclusion Chromatography. *Langmuir* **40,** 8365–8372. issn: 0743-7463 (Apr. 2024).

13. Chau, C., Mohanan, G., Macaulay, I., Actis, P. & Wälti, C. Automated Purification of DNA Origami with SPRI Beads. *Small* **20.** issn: 1613-6810 (May 2024).

14. Wagenbauer, K. F. *et al.* How We Make DNA Origami. *ChemBioChem* **18,** 1873–1885. issn: 1439-4227 (Oct. 2017).

15. M. Scheckenbach, G. A. Brüggenthies, T. Schröder, K. Betuker, L. Wassermann, P. Tinnefeld, A. Heuer-Jungemann, V. Glembockyte, Monitoring the Coating of Single DNA Origami Nanostructures with a Molecular Fluorescence Lifetime Sensor. *Small* 2025, 21, 2501044. https://doi.org/10.1002/smll.202501044

8 Addendum

8.1 AFM pre/post purification Streptavidin conjugation

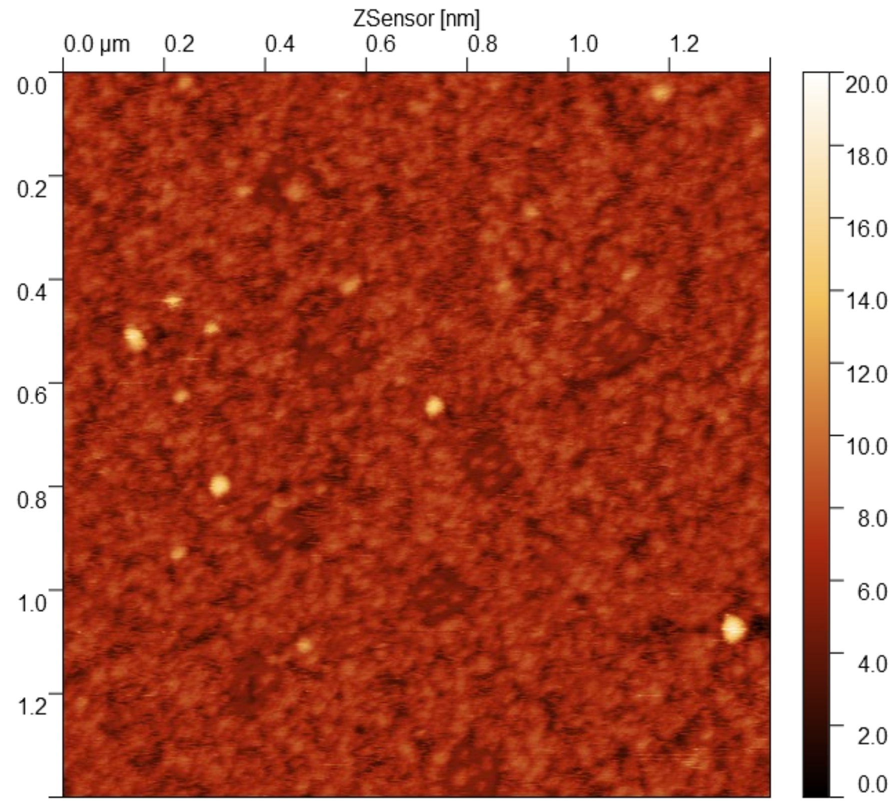

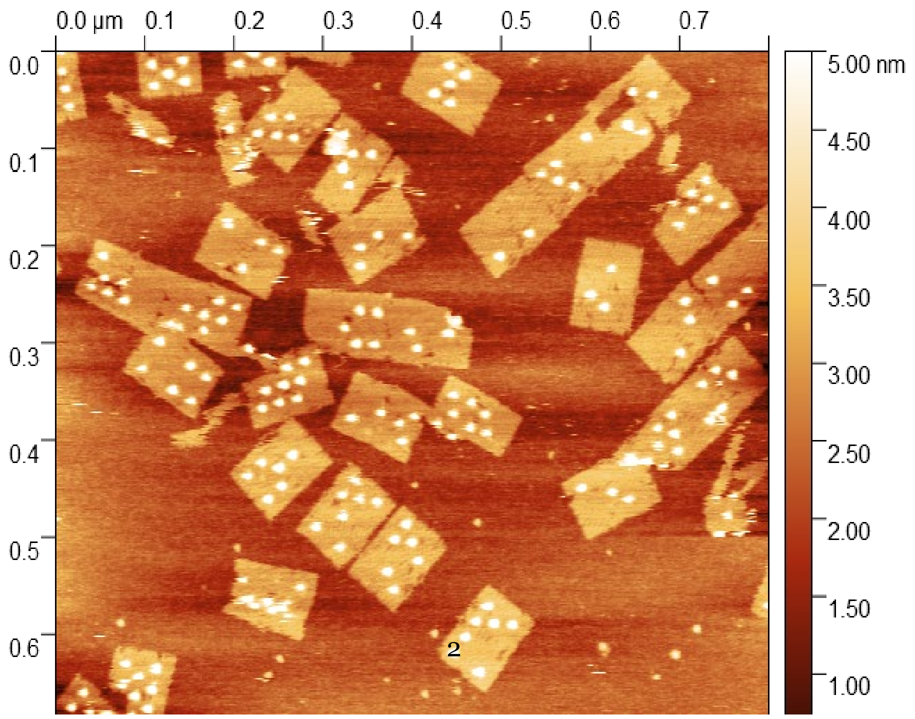

FigureS19 DNA origami conjugated with streptavidin before (top) and after purification (Fraction 3) (bottom).

8.2 Uncropped Gel images for Figure 4

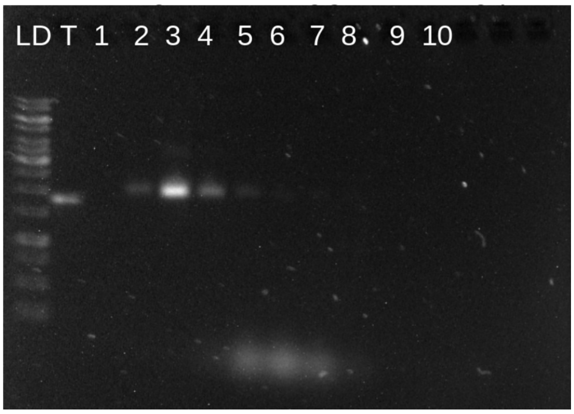

Figure S20: 0.8mL CV SuperSEC with 50μL Rectangle DNA Origami, 100 μL fractions

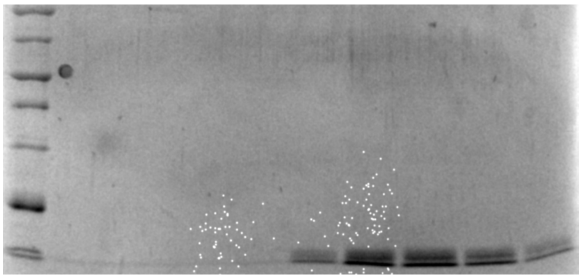

Figure S21: 0.8mL CV SuperSEC SDS-PAGE, 50μL 16.7μM Streptavidin, 100μL Fractions.

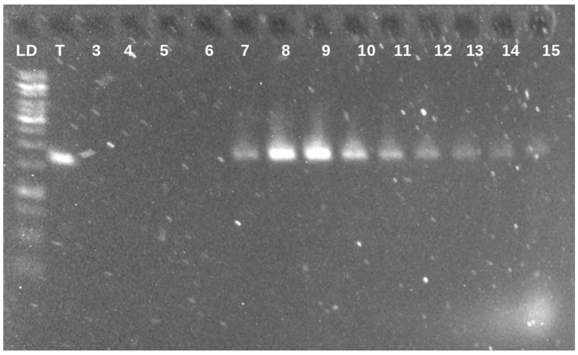

Figure S22: 2.5mL CV SuperSEC with 50μL Rectangle DNA Origami, 125 μL fractions

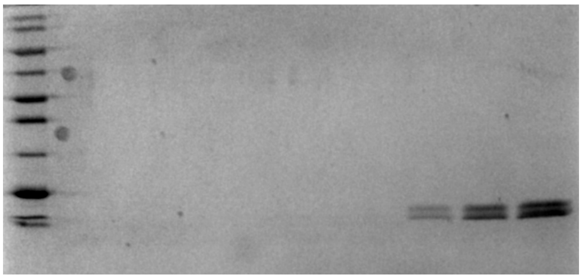

Figure S23: 2.5mL CV SuperSEC SDS-PAGE, 50μL 16.7μM Streptavidin, 250μL Fractions.

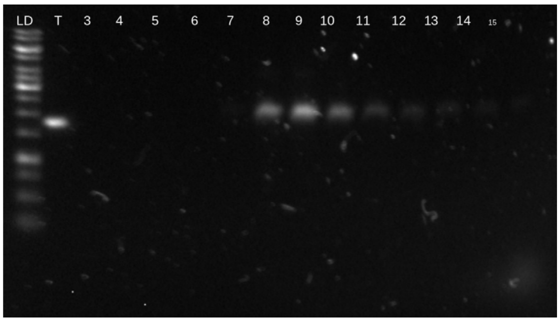

Figure S24: 5mL CV SuperSEC with 50μL Rectangle DNA Origami, 250 μL fractions

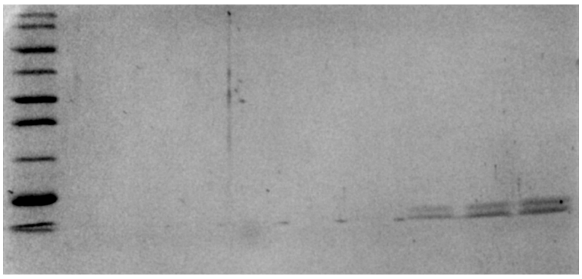

Figure S25: 5mL CV SuperSEC SDS-PAGE, 50μL 16.7μM Streptavidin, 500μL Fractions.

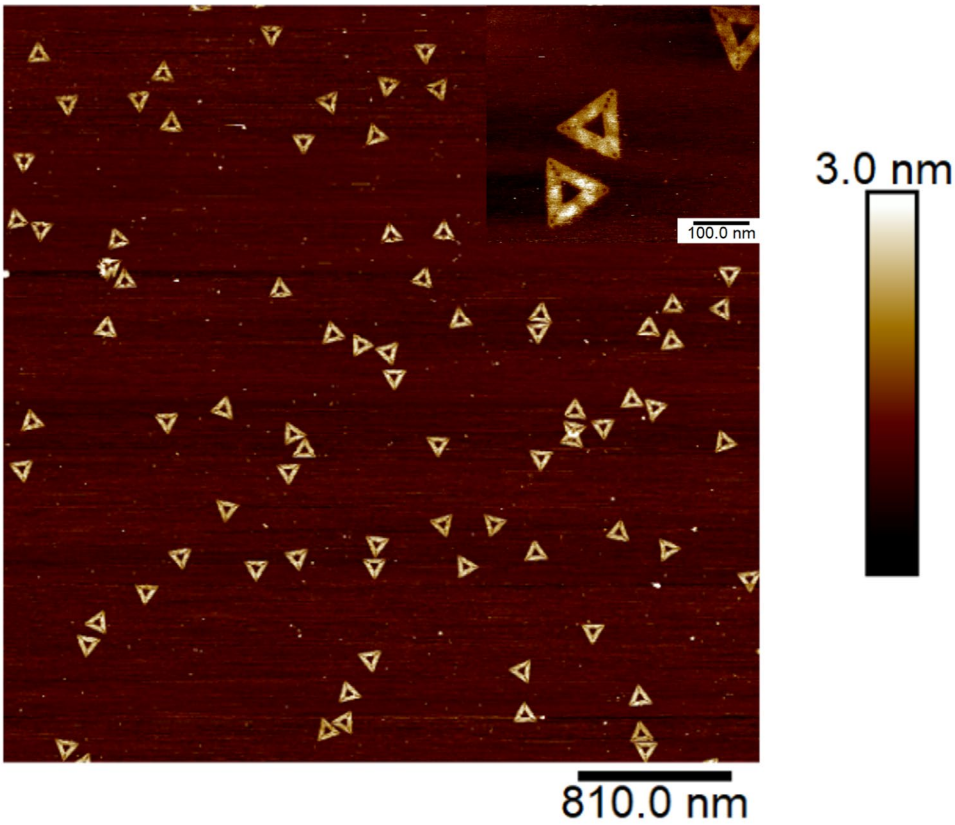

Figure S26: Low magnification image of TBP-functionalised nanostructures, and high magnification inset on the top right corner.

8.3 Intactness counting images

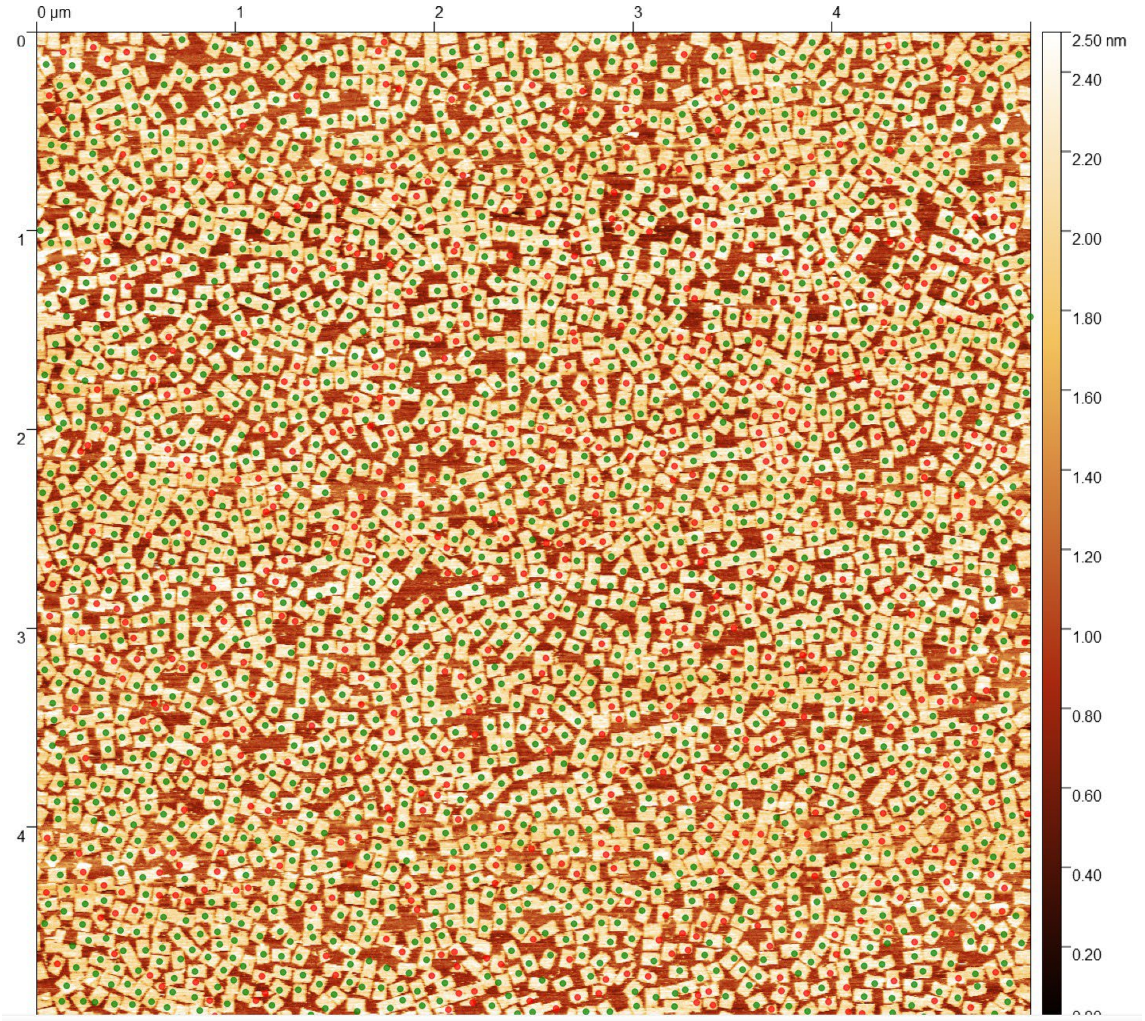

Figure S27: Rectangle unpurified 5x5μm AFM image

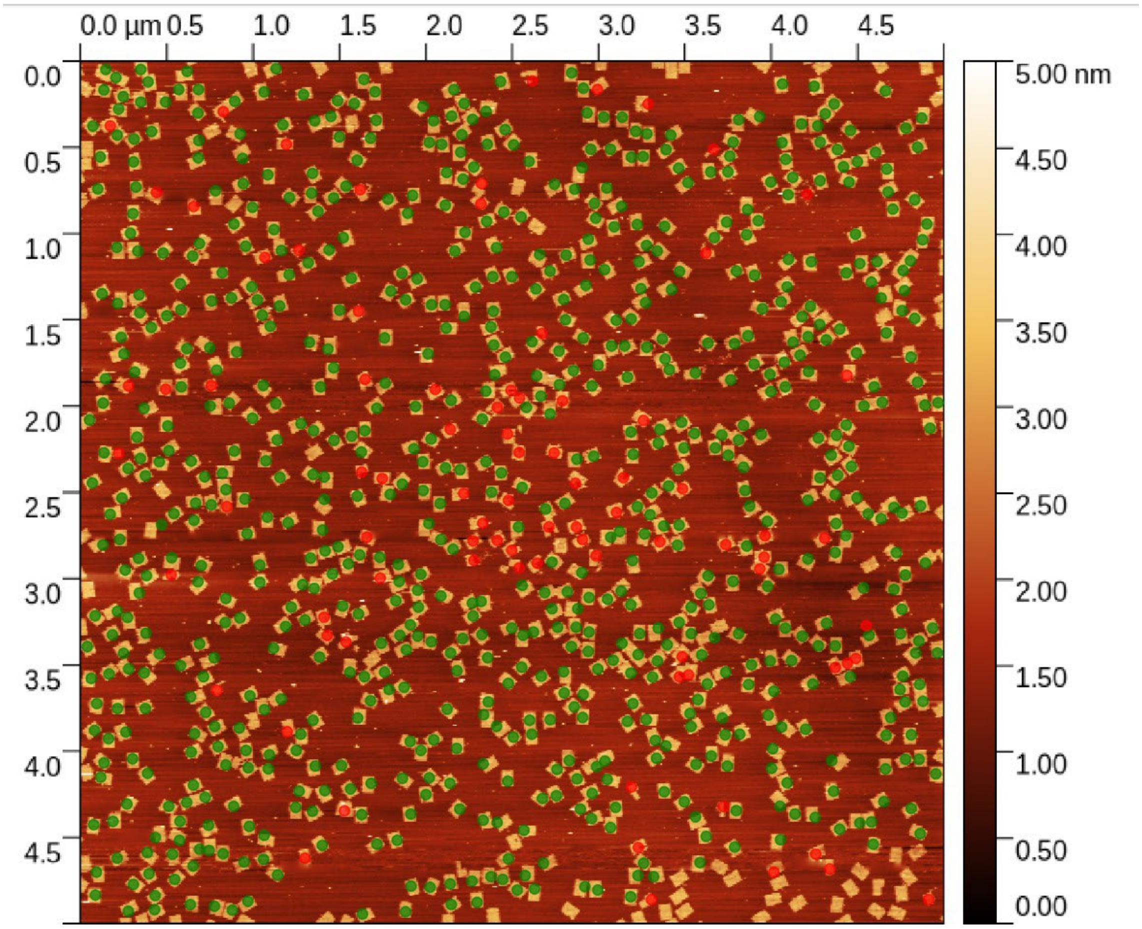

Figure S28: Rectangle purified Fraction 3 (300μL EV)5x5μm AFM image

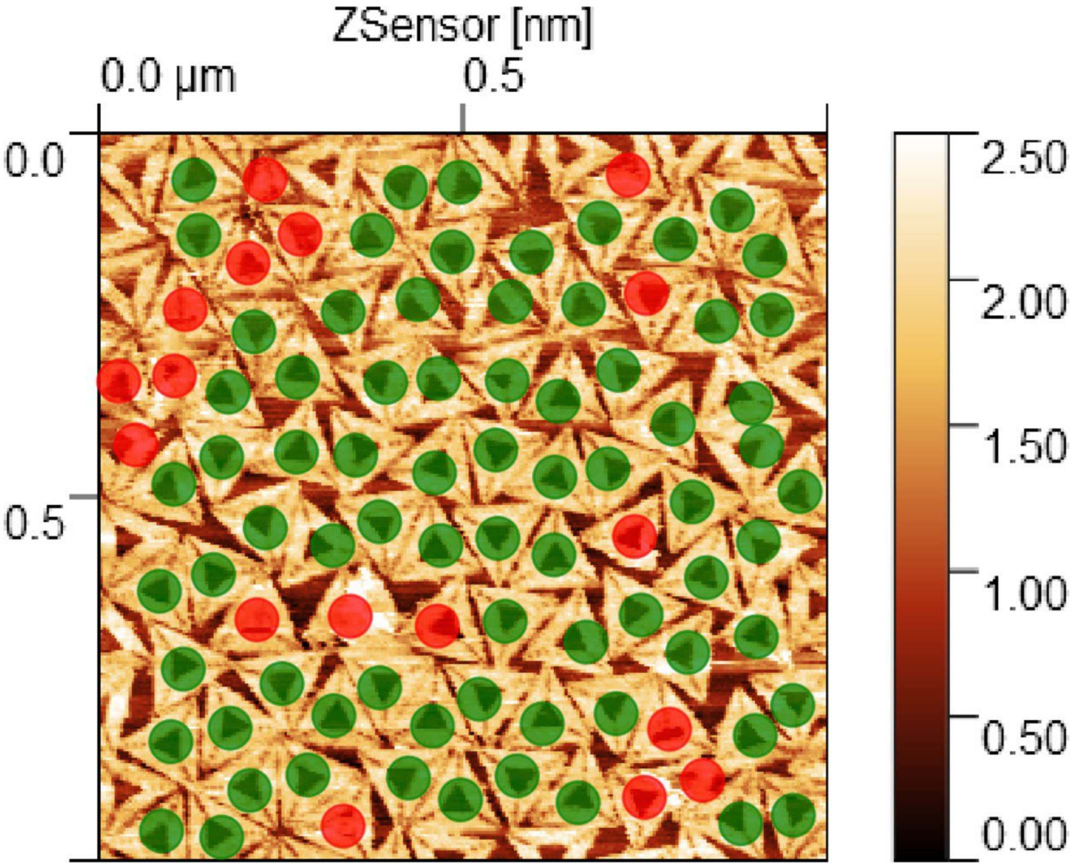

Figure S29: Triangle unpurified 5x5μm AFM image

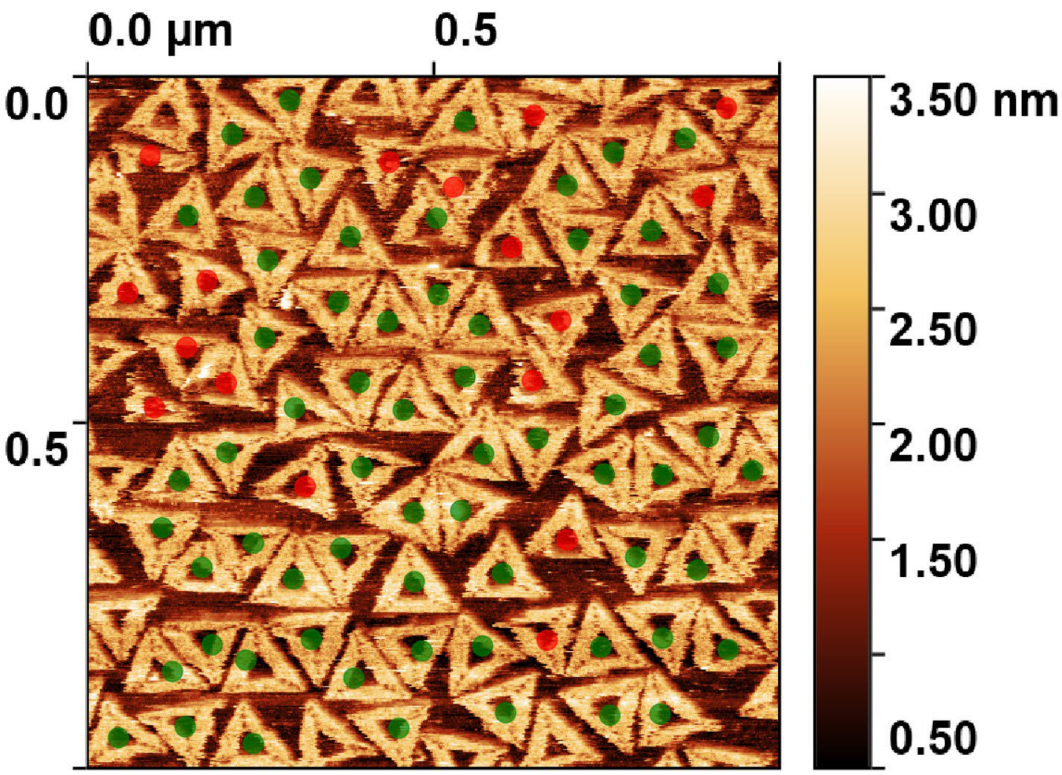

Figure S30: Triangle purified Fraction 3 (300μL EV)5x5μm AFM image

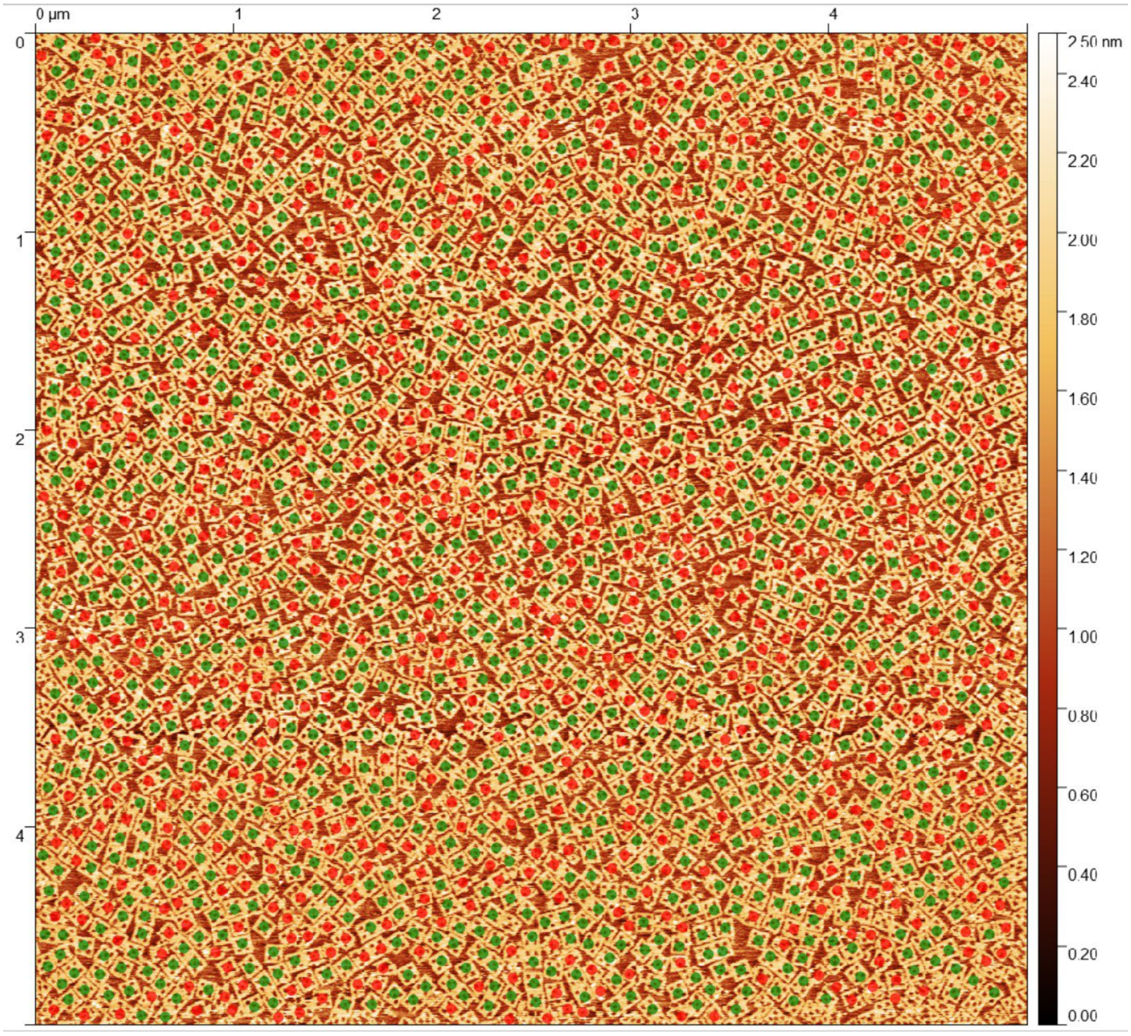

Figure S31: 5-well Frame unpurified 5x5μm AFM image

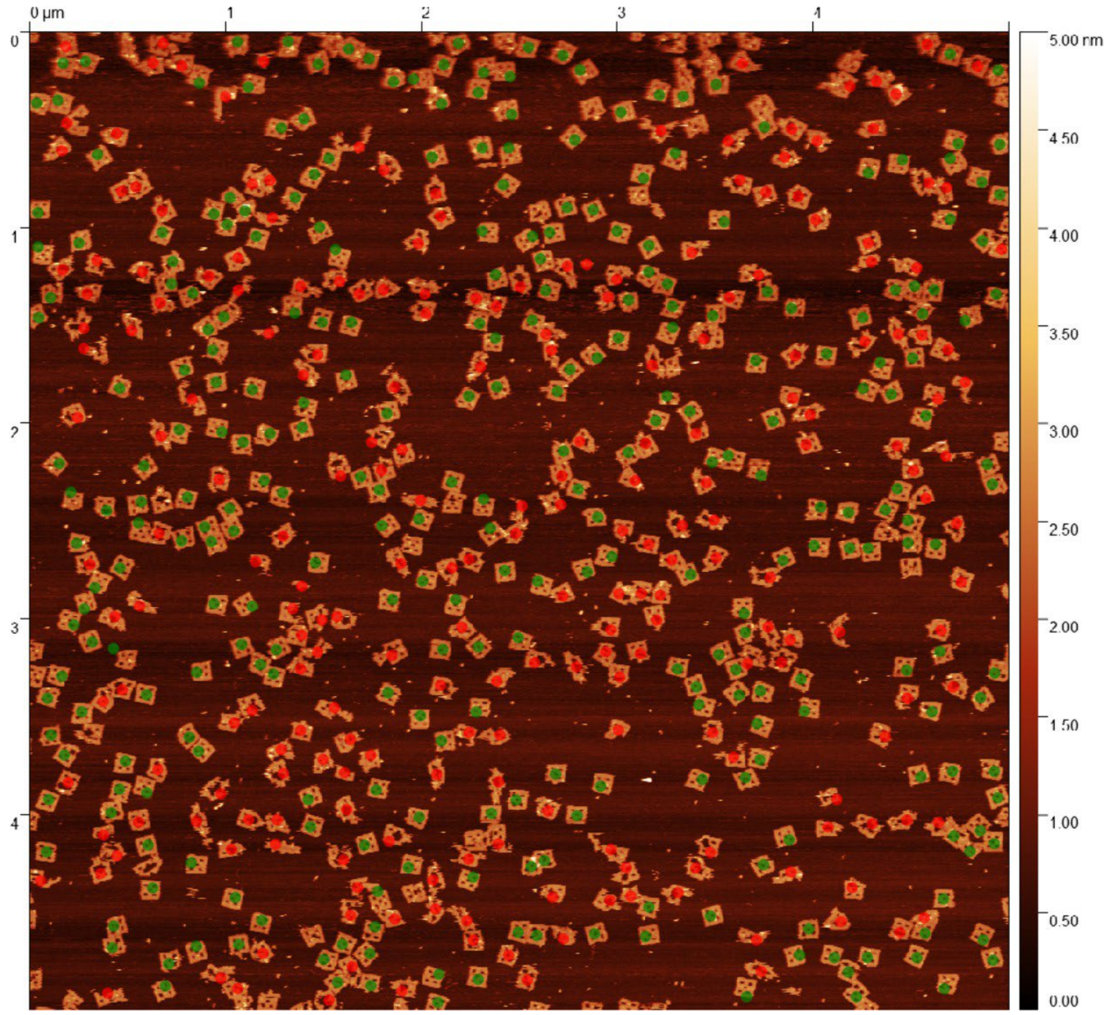

Figure S32: 5-well Frame purified Fraction 3 (300μL EV)5x5μm AFM image

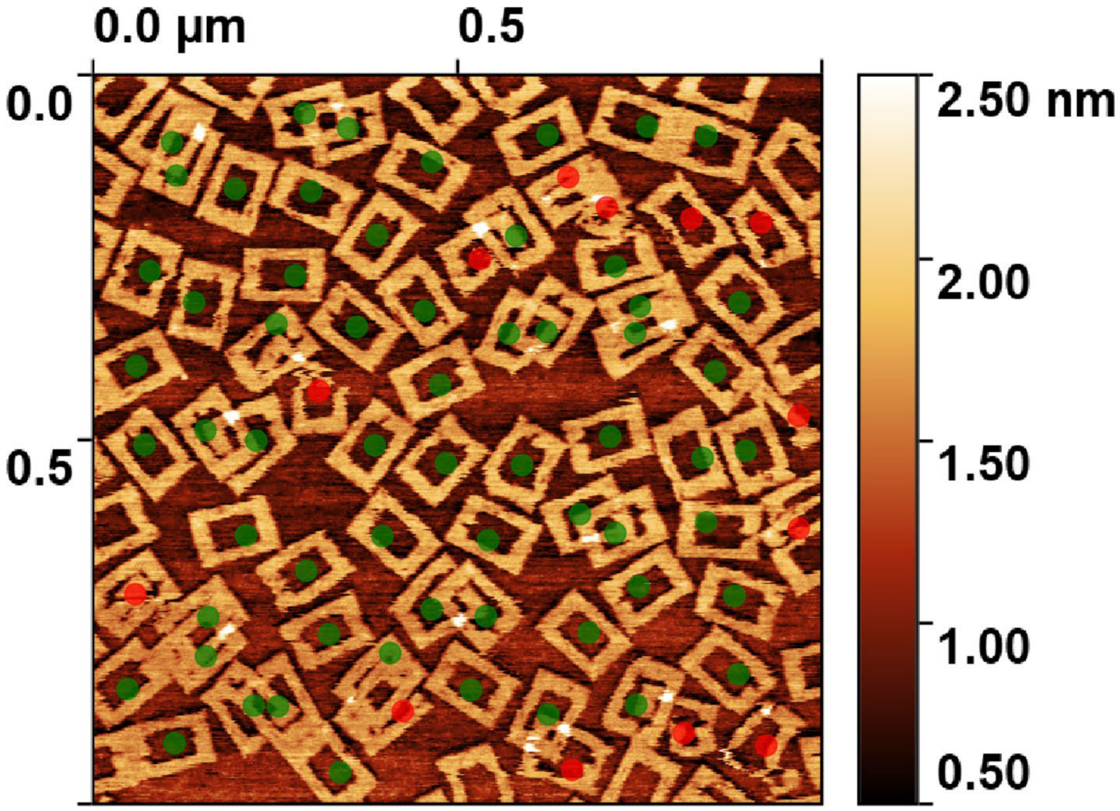

Figure S33: Frame unpurified 5x5μm AFM image

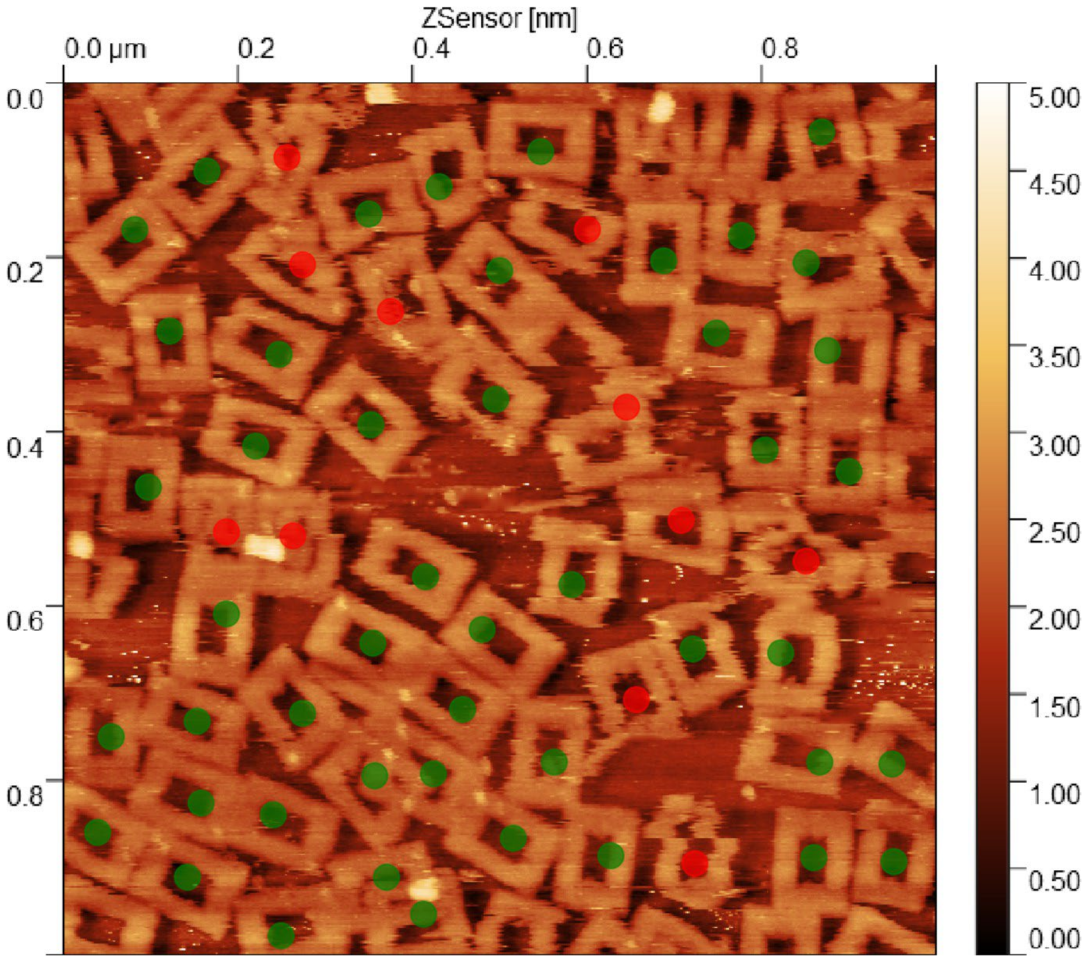

Figure S34: Frame purified Fraction 3 (300μL EV)5x5μm AFM image
